# Genome-wide discovery of somatic regulatory variants in diffuse large B-cell lymphoma

**DOI:** 10.1038/s41467-018-06354-3

**Published:** 2018-10-01

**Authors:** Sarah E. Arthur, Aixiang Jiang, Bruno M. Grande, Miguel Alcaide, Razvan Cojocaru, Christopher K. Rushton, Anja Mottok, Laura K. Hilton, Prince Kumar Lat, Eric Y. Zhao, Luka Culibrk, Daisuke Ennishi, Selin Jessa, Lauren Chong, Nicole Thomas, Prasath Pararajalingam, Barbara Meissner, Merrill Boyle, Jordan Davidson, Kevin R. Bushell, Daniel Lai, Pedro Farinha, Graham W. Slack, Gregg B. Morin, Sohrab Shah, Dipankar Sen, Steven J. M. Jones, Andrew J. Mungall, Randy D. Gascoyne, Timothy E. Audas, Peter Unrau, Marco A. Marra, Joseph M. Connors, Christian Steidl, David W. Scott, Ryan D. Morin

**Affiliations:** 1 Department of Molecular Biology and Biochemistry, Simon Fraser University, Burnaby, BC V5A 1S6 Canada; 2 Lymphoid Cancer Research, British Columbia Cancer Research Centre, Vancouver, BC V5Z 1L3 Canada; 3 Canada’s Michael Smith Genome Sciences Centre, British Columbia Cancer Agency, Vancouver, BC V5Z 1L3 Canada; 4 Molecular Oncology, British Columbia Cancer Research Centre, Vancouver, BC V5Z 1L3 Canada; 5 Department of Medical Genetics, University of British Columbia, Vancouver, BC Canada

## Abstract

Diffuse large B-cell lymphoma (DLBCL) is an aggressive cancer originating from mature B-cells. Prognosis is strongly associated with molecular subgroup, although the driver mutations that distinguish the two main subgroups remain poorly defined. Through an integrative analysis of whole genomes, exomes, and transcriptomes, we have uncovered genes and non-coding loci that are commonly mutated in DLBCL. Our analysis has identified novel *cis*-regulatory sites, and implicates recurrent mutations in the 3′ UTR of *NFKBIZ* as a novel mechanism of oncogene deregulation and NF-*κ*B pathway activation in the activated B-cell (ABC) subgroup. Small amplifications associated with over-expression of *FCGR2B* (the Fc*γ* receptor protein IIB), primarily in the germinal centre B-cell (GCB) subgroup, correlate with poor patient outcomes suggestive of a novel oncogene. These results expand the list of subgroup driver mutations that may facilitate implementation of improved diagnostic assays and could offer new avenues for the development of targeted therapeutics.

## Abstract (web summary)

The driver mutations for the two main molecular subgroups of diffuse large B-cell lymphoma (DLBCL) are poorly defined. Here, an integrative genomics analysis identifies 3′ UTR *NFKBIZ* mutations within the activated B-cell DLBCL subgroup and small *FCGR2B* amplifications in the germinal centre B-cell DLBCL subgroup.

## Introduction

It has been established that DLBCL, although genetically heterogeneous, can be robustly divided at the gene expression level into two “cell of origin” (COO) subgroups based on markers of B-cell differentiation and NF-*κ*B activity pathways, where high NF-*κ*B activity is a hallmark of the ABC subgroup^1^. *EZH2*^2^, *SGK1*, *GNA13* and *MEF2B*^2^ exemplify genes that are mutated exclusively in GCB cases, whereas mutations in *MYD88*^3^, *CD79B*^4^ and *CARD11*^5^ are reportedly more common in ABC. Some DLBCL cases have few mutations that are characteristic of either subgroup, suggesting that additional genetic changes may shape the malignancy. Similarly, the over-expression of proteins with potential therapeutic and clinical relevance cannot always be explained by known genetic alterations^6^. Gaining a more complete understanding of the genetic features of DLBCL in general, and each subgroup in particular, should lead to improved methods for sub-classification, and further inform on the molecular and genetic underpinnings of the lymphoma found in individual patients. Such enhancements have the potential to facilitate the development of therapies such as small molecule inhibitors^7^ or monoclonal antibodies and immunotherapies that target somatic mutations or cell surface proteins^8^.

Although there have now been thousands of DLBCL tumours analysed using targeted strategies such as array-based copy number analysis^9^ or whole exome sequencing (WES)^10^, a limited number of complete DLBCL genomes have been described to date^11–13^. Nonetheless, further analysis of DLBCL using whole genome sequencing (WGS) has significant potential to uncover new somatic structural variations (SVs), copy number alterations (CNAs) and other *cis*-acting regulatory mutations that may be cryptic to more targeted approaches. In several lymphoid cancers, including DLBCL, the enzyme AID (encoded by *AICDA*), in cooperation with POL*η*, induces mutations in actively transcribed genes through the process of aberrant somatic hypermutation (aSHM)^14^, which affects a substantial number of loci in these cancers relative to other B-cell lymphomas^15^. As the repertoire of known aSHM targets in lymphoma continues to grow, it has become apparent that this process can also impact non-genic loci associated with super-enhancers. Given the disproportionate representation of mutations in non-coding regions, a thorough evaluation of the potential for regulatory driver mutations in aSHM targets and elsewhere is warranted^16,17^.

Here, we present a novel strategy to identify coding and non-coding regions with an enrichment of somatic mutations genome-wide in large cohorts of patients, allowing us to identify sites affected by aSHM or with clustered mutations resulting from positive selection and infer their potential *cis*-regulatory effects on coding genes^11,12^. We analysed WGS data from 153 DLBCL tumour/normal pairs (discovery cohort), perform validation on an additional 338 cases (internal validation cohort) and compare these results to existing WES data from over 1000 additional cases (external validation cohort)^10^ to identify coding and non-coding loci recurrently affected by somatic single nucleotide variants (SNVs) or indels, collectively referred to as simple somatic mutations (SSMs) in DLBCL. Through the analysis of matched RNA-seq data, we uncovered the effect of recurrent structural variations (SVs) and recurrently mutated non-coding regions in mediating the transcriptional or post-transcriptional regulation of numerous genes with relevance to DLBCL.

## Results

### Local mutation density of SSMs

**Fig. 1:**
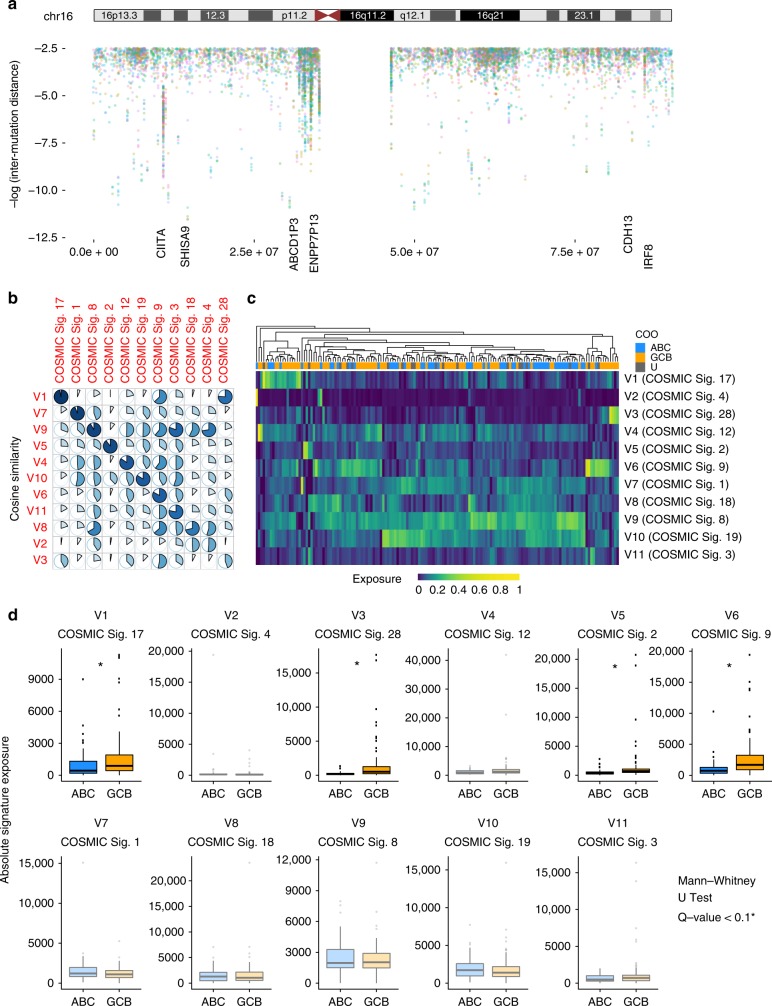
Rainstorm and mutation signature analysis of DLBCL genomes. **a** An overview of mutation peaks and the rainstorm representation of cohort-wide inter-mutation distance for chromosome 16. Peaks identified by the Doppler algorithm that could be attributed to a nearby gene are labelled below. Known aSHM targets such as *CIITA* and *IRF8* are among the most visible peaks in the Rainstorm view. **b** Our de novo inference of mutation signatures from the entire cohort revealed 11 robust signatures. Each signature was assigned to a reference signature from the curated set of 30 signatures in the Catalogue of Somatic Mutations in Cancer (COSMIC) database based on cosine similarity. The individual pie charts represent the strength of this similarity. The rows are arranged such that those with weaker similarity to a known signature are near the bottom. **c** A heat map showing the exposure of all 11 signatures in the genomes. Cases (columns) and signatures (rows) are ordered based on hierarchical clustering on the relative exposures. **d** Comparison of the exposure for the signatures in GCB and ABC cases including the four signatures with significantly higher exposure in GCB cases (indicated with an asterisk). The lower, middle and upper boxplot hinges correspond to the 25th, 50th and 75th percentiles, respectively. The boxplot whiskers extend outwards past the hinges up to the inter-quartile range×1.5 or the farthest value, whichever is closest

In each of the 153 paired DLBCL genomes (cohort details in Supplementary Data 1), we detected between 1,689 and 121,694 SSMs (median: 14,026; Supplementary Data 2). We separately inferred somatic copy number variations (CNVs) and 12,609 structural variation (SV) breakpoints (range: 0–390; median 66; Supplementary Data 3) and annotated these based on proximity to genes. We implemented two new algorithms, Rainstorm and Doppler, that infer regions of arbitrary span with SSM density elevated above the local background. Rainstorm considers the positions of mutations pooled from a cohort of cancer genomes (optionally excluding any variants within the coding region of genes) and calculates local mutation density relative to each mutation, similar in principle to rainfall plots^18^. Doppler then infers the presence and boundaries of peaks of elevated local mutation rate. An initial analysis that excluded all mutations in coding regions detected 4,386 such peaks among the discovery cohort ranging from a single nucleotide to many kilobases (kb) in length (median length: 664 nucleotides; Fig. 1a; Supplementary Data 4). The regions within these peaks exhibited a median mutation density of 10.3 per kb, whereas a randomly selected region showed, on average, 1.00 mutation per kb. Our analysis also revealed examples of non-coding loci with mutation peaks, for example the two adjacent long non-coding RNA (lncRNA) genes *NEAT1* and *MALAT1* and the microRNA miR-142. Mutations at each of these loci have been previously noted in DLBCL and FL with a pattern consistent with aSHM^19,20^.

To determine the suitability of our approach to identify loci with mutations relevant to DLBCL biology, we applied Rainstorm/Doppler to all mutations including those within coding regions. We found a similar number of peaks (4,405), which comprised the bulk of original regions along with peaks in genes with known mutation hot spots such as *EZH2*, *FOXO1,* and *MYD88* (Supplementary Data 5). Aside from intergenic regions (2,214), the top three peak annotations were Intron (1,620), 5′ Flank (258) and 3′ Flank (208). These are also the regions typically affected by aSHM and, as expected, virtually all of the known targets of aSHM^12,15^ were represented among the Doppler peaks. Some genes recurrently affected by non-silent mutations in DLBCL also displayed an excess of mutations affecting their non-coding regions, including *SGK1*, *PRDM1*, *TMSB4X,* and *TBL1XR1*.

The relative representation of SNVs affecting distinct trinucleotide contexts, known as mutation signatures, can inform on the major mutational processes in a tumour. Using standard methods^21^, we inferred a robust set of 11 de novo signatures from the entire cohort and assigned each to a COSMIC reference signature on the basis of cosine similarity (Fig. 1b; Supplementary Figure 2). Hierarchical clustering of the cases based on the relative abundance of each signature (“exposure”) did not recapitulate the molecular subgroups (Fig. 1c), though a direct comparison between ABC and GCB cases revealed four signatures with significantly higher exposure among GCB cases (Wilcoxon rank-sum test, *P* < 0.05) (Fig. 1d). These include V6, a signature closely resembling one attributed to AID-mediated SHM (COSMIC Signature 9), which was identified in lymphoid cancers^21,^ and V2, one of the more unique signatures identified herein (Supplementary Figure 2). Given that AID is a cytidine deaminase, we compared the proportion of mutations affecting the C (or G) within AID recognition motifs that fall within and outside peaks and confirmed a significant enrichment of mutations in this context within the Doppler peaks (*P* < 2.2 × 10^−16^, Fisher’s exact test). Although this points to AID activity as a major process driving mutagenesis in DLBCL, there is clearly a variable collection of other mutagenic processes at play. ABC cases showed lower exposure to the AID-related signature, though there were ABC cases with mutations in some of the peaks attributed to known aSHM targets. Paradoxically, the expression of AID was significantly higher among the ABC cases in our internal validation cohort (*P* = 9.1 × 10^−6^, Wilcoxon rank-sum test). There was also substantial variability in the exposure to this signature within GCB genomes. Taken together, these data suggest that other biological variables beyond COO affect the extent of AID-mediated mutation and the specific loci targeted by this process in DLBCL.

### Identifying candidate *cis*-regulatory mutations

The predominant mutation type known to directly affect gene expression in *cis* in DLBCL are translocations and other SVs. As expected, genes most frequently proximal to SVs were oncogenes with known relevance in DLBCL including *BCL2*, *BCL6*, *FOXP1,* and *MYC* (Supplementary Figure 3). Some SVs affecting known or suspected oncogenes appeared within the gene body, such as those in *FOXP1*^22^, *TBL1XR1,* or *NFKBIZ*, which can lead to novel isoforms or fusion transcripts^23^. We searched for putative *cis*-regulatory variation by comparing the proximity of SVs to CNV foci previously identified through analysis of our validation cohort (Table 1; Ennishi et al., unpublished). Tumour suppressor genes (TSGs) more commonly contained SV breakpoints (typically deletions) within the gene body, including *TP53*, *CDKN2A*, and *CD58*. Some loci affected by a combination of SVs and CNVs also had nearby Doppler peaks (e.g. *MEF2B* and *NFKBIZ*; Table 1). In contrast, *TOX* and *WWOX* harboured a substantial number of distinct breakpoints including several examples of focal deletions but rarely contained SSMs (Supplementary Figure 4A). However, few patients harboured SVs in *TOX* and *WWOX*, indicating these genes may rarely act as tumour suppressor genes in DLBCL. Many of the known aSHM targets were also enriched for SVs including *MEF2B*, a gene with multiple known mutation hot spots, particularly in GCB DLBCL. The function of *MEF2B* mutation in DLBCL has not been definitively established^24,25,^ and these putative inactivating mutations provides further evidence of its role as a tumour suppressor but does not eliminate the possibility of shortened isoforms with an enhanced or distinct activity. Further complicating matters, *MEF2B* SVs were predominantly found in ABC, whereas hot spot mutations are a known feature of GCB, possibly indicating distinct roles of this gene in each subgroup.

**Table 1:** Overview of SVs and CNVs proximal to genes detected by WGS

	Structural Variation				Recurrent CNV			Summary	
	Del	Tra	Dup	Inv	Num (type)	Median	Minimum	Total	Doppler Peak?
*TCF4*	5	2	2	1	41 (A)	12986372	73803	44	no
*CDKN2A*	22	20	0	1	22 (D)	16505508	400124	42	none
*NFKBIZ*	6	3	0	3	31 (A)	17720083	944075	36	3′ UTR^a^
*FOXP1*	9	6	2	0	27 (A)	19034690	3207496	35	intron^a^
*FCGR2B*	2	0	0	2	33 (A)	11049954	96085	34	no^a^
*IKBKE*	1	0	1	0	28 (A)	15176955	1095013	29	no
*CD58*	14	10	4	0	11 (D)	8488587	559852	25	introns^b^
*TOX*	12	8	2	1	10 (D)	35182055	192657	22	no
*CIITA*	13	9	1	3	7 (D)	6536287	1151750	20	intron^a^
*TP53*	4	2	0	0	18 (D)	9410568	1145996	21	none^b^
*MEF2B*	10	9	0	1	8 (D)	7855612	1863130	18	none^b^
*ETV6*	10	8	2	1	3 (D)	19441596	3190056	13	intron 1
*IRF8*	4	2	1	1	3 (D)	7701889	185094	7	intron 1^b^
*BCL2L11*	5	5	1	0	2 (D)	7321203	339970	7	intron 1

SVs are separately counted by the type of event as determined by read pairing information. The total number of CNVs in the direction associated with the recurrent alteration (A or D) and the median and minimum of these is shown to highlight the focal nature of some of these events

Tra, translocation; Del, deletion; Dup, duplication; Inv, inversion; A, copy number amplification or gain; D, copy number deletion

^a^ Region was subjected to targeted sequencing to determine prevalance of coding and non-coding mutations

^b^Region was subjected to targeted sequencing to determine prevalence of coding mutations

We utilised RNA-seq-derived expression values from a subset of the discovery cohort cases to infer *cis* effects of these events on expression. Through this analysis, both *NFKBIZ* and *FCGR2B* were identified as candidate oncogenes based upon significantly elevated expression in cases with either a gain or proximal SV (Supplementary Figure 4B). *NFKBIZ* has been reported as a target of amplification in some DLBCLs but has not, to our knowledge, been shown to be deregulated through SVs^26^. We extended this analysis to identify Doppler peaks with potential relevance in modulating transcription by determining peaks whose mutation status was associated with the expression of nearby genes (Supplementary Figure 5). Most protein-coding loci whereby expression correlated with mutation status were known targets of aSHM (including *SERPINA9*, *CD44*, and *PIM1*) or novel targets identified herein (including *DNMT1* and *AICDA*). However, there are many additional genes with high expression levels that did not appear to be influenced by aSHM, demonstrating that expression alone is insufficient to explain aSHM. Nonetheless, this subset of genes that are affected by aSHM may act as a permanent record indicating sustained or past high gene expression and thereby a genetic marker of their cell of origin. Although the bulk of these may therefore not represent driver mutations, the unprecedented breadth of mutations affecting potential regulatory regions including enhancers proximal to these genes suggests the potential for some to affect gene expression and thus warrants further investigation.

### Recurrently mutated loci associated with ABC or GCB DLBCL

**Fig. 2:**
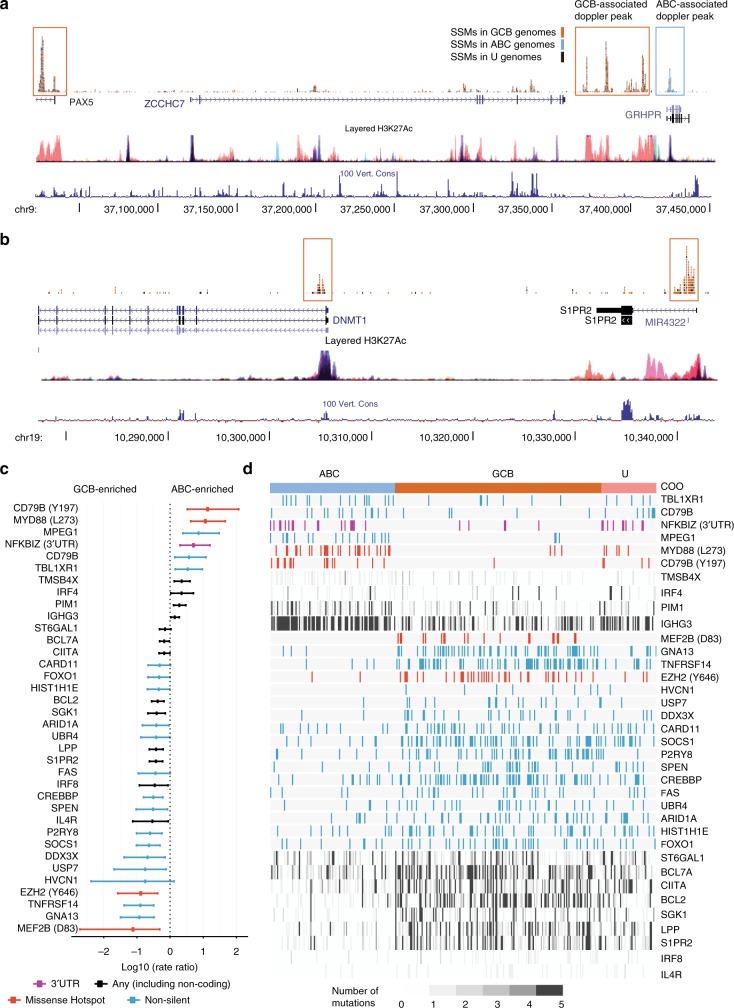
Differences in mutational representation between DLBCL molecular subgroups. **a** An enhancer proximal to *PAX5* was preferentially mutated in GCB cases. A nearby peak in *GRHPR* near *PAX5* was more commonly mutated in ABC cases. Non-coding mutation of the enhancer proximal to *PAX5* has been reported in CLL but has not, to our knowledge, been described in other lymphoid cancers. The mutation pattern in DLBCL resembles that of other super-enhancers (Supplementary Figure 6B). **b** *S1PR2* is a known target of aSHM, and the mutations mainly affect the first intron. *DNMT1* is adjacent to *S1PR2* and has a similar mutation pattern. Both of these peaks were enriched for mutations in GCB, indicating the potential for co-regulation of these genes using a common set of regulatory regions. **c** Coding and non-coding mutations that may be associated with either ABC or GCB COO are shown based on our recurrence cohort and are ordered on the strength of the association. For genes with missense mutation hot spots or (for *NFKBIZ*) a 3′ UTR hot spot, only mutations affecting that region were considered (indicated in parentheses beside the gene). Either hot spot, coding, or all mutations were used for this calculation, depending on the gene, as indicated in the legend. **d** The mutations detected in these genes are shown for each patient in our validation cohort. For genes affected by aSHM, mutations are represented using grey scale to indicate the number of mutations detected in each patient

By comparing mutation abundance within peaks derived from the full set of mutations, we identified 89 sites significantly enriched for mutations in either ABC (37) or GCB (52) cases (Supplementary Figure 6A; Supplementary Data 6). The bulk of mutations in many of these loci affected introns, the 5′ UTR or upstream of the TSS, and unsurprisingly, many were known aSHM targets discussed above (Fig. 2a; Supplementary Figure 7). Some hypermutated loci contained multiple discrete peaks. For example, the *BCL6* locus and its nearby super-enhancer contained 31 discrete peaks (Supplementary Figure 6B). We also noted a second mutation peak in the intron of *BCL2* distal to the TSS that appears to be a regulatory region (Supplementary Figure 6C).

**Fig. 3:**
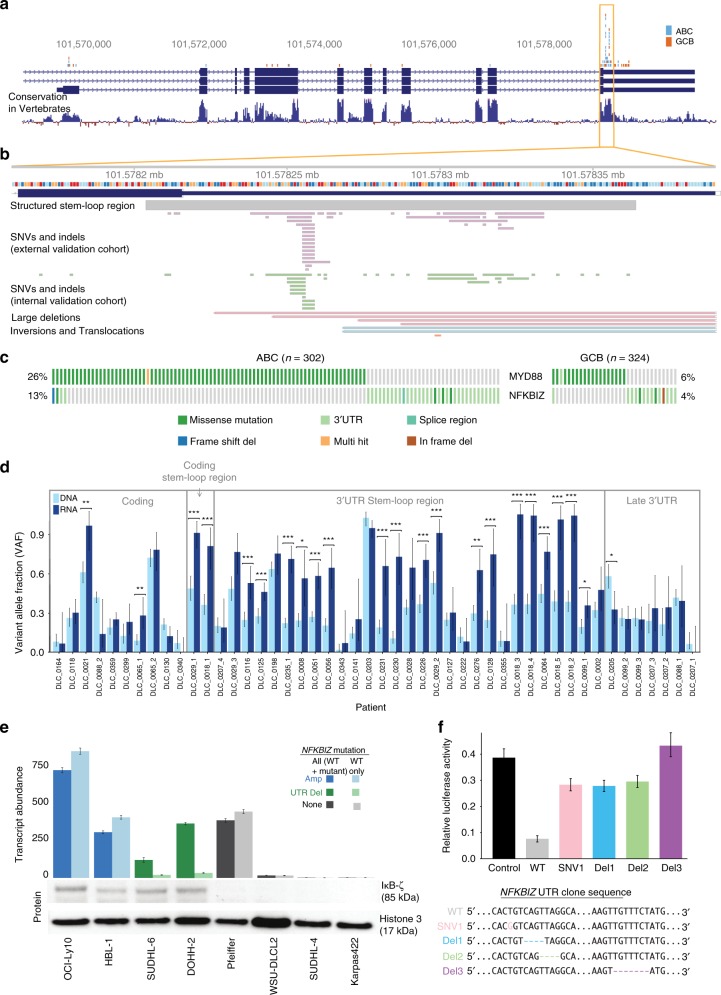
Mutations affecting the *NFKBIZ* locus and functional effects on mRNA and protein levels. **a** *NFKBIZ* mutations were predominantly found within a highly conserved region of the 3′ UTR and were significantly enriched in ABC cases (blue) relative to GCB cases (orange). **b** A detailed view of the mutated region including the location predicted to have conserved structure (in grey). The pattern of mutations is similar in both the internal validation cohort (322 cases) and the external validation cohort (984 cases). **c** Mutations in *NFKBIZ* and *MYD88* within ABC and GCB cases in the larger external validation cohort. The same trend of mutual exclusivity was observed in both validation cohorts. **d** Comparison of mutant variant allele fractions (VAFs) from DNA sequencing and RNA-seq of patient samples with *NFKBIZ* mutations. VAFs higher in RNA relative to the corresponding DNA indicates allelic imbalance favouring the mutant allele. Significant differences are indicated (*P < 0.05, **P < 0.01, ***P < 0.001, Wilcoxon rank-sum test). **e** We applied a custom ddPCR assay to eight DLBCL cell lines to determine *NFKBIZ* mRNA expression levels. Mutant cell lines consistently showed increased *NFKBIZ* mRNA, and we could attribute this to the mutant allele in lines with 3′ UTR mutations (green). Cell line I*κ*B-*ζ* expression was assessed by western blot. Only mutant cell lines (green and blue) showed increased protein. **f** Luciferase reporter assay results show reduced protein expression in the presence of wild-type UTR with restored expression in mutant constructs. Luciferase expression is normalised to a construct containing a latter portion of the UTR. Error bars represent s.d. from three replicates

We tested each of the COO-associated peaks for association with treatment outcome in the discovery cohort using univariate Kaplan-Meier models. We identified a significant association between each of *CIITA* and *IGHG1* mutation status and shorter time to progression (TTP) and disease-specific survival (DSS) but these did not retain significance after correction for multiple hypothesis testing. Using our internal validation cohort (Supplementary Table 1), we performed targeted sequencing on both the coding regions for a large set of known DLBCL-related genes and genes identified from this analysis as enriched for non-silent mutations (Supplementary Table 7) along with a selection of these non-coding peaks. Of those selected for validation, we confirmed 10 loci were enriched for mutations in ABC and 26 were enriched in GCB (Fig. 2c, d). In contrast to prior studies, *CARD11* mutations were found here to be significantly enriched in GCB cases. The four sites with the strongest specificity for ABC were *CD79B* (Y197), *MYD88* (L273), *MPEG1,* and the 3′ UTR of *NFKBIZ* (Fig. 2; Supplementary Figure 6). The majority of mutations affecting *CIITA*, *IGHG*, and *NFKBIZ* were non-coding and, with the exception of *NFKBIZ*, were consistent with being aSHM targets (Supplementary Figure 7A). *NFKBIZ* mutations were almost entirely within the 3′ UTR, and most did not affect AID motifs (Fig. 3a). In our external validation cohort, we found a nearly identical pattern of SSMs in the *NFKBIZ* 3′ (Fig. 3b), and within the ABC sub-type, mutations in *NFKBIZ* and *MYD88* were significantly mutually exclusive (*P* = 0.0042, CoMEt exact test). We determined the prevalence of this mutation in other lymphoid cancers with available WGS data including CLL, FL, and BL. FL had the next highest prevalence of *NFKBIZ* 3′ UTR mutations mutations appearing in <3% of cases, suggesting these mutations are specific to DLBCL.

### Functional characterisation of *NFKBIZ* 3′ UTR variants

The specificity of *NFKBIZ* 3′ UTR mutations to DLBCL (particularly ABC cases) suggests a strong selective pressure and implicates them as having a regulatory role in *cis* (Fig. 3). The mutated region is highly conserved and has been predicted to form multiple stable secondary structures which are thought to contain a destabilising element that promotes rapid mRNA turnover^27,28^. Through available DLBCL cell line WGS data^11^, we identified 3′ UTR mutations in two cell lines (DOHH-2 and SU-DHL-6) and amplification of this locus in two additional lines (OCI-Ly10 and HBL-1). *NFKBIZ* mRNA levels were consistently higher among cases with 3′ UTR mutations or amplifications, supporting a common role in promoting *NFKBIZ* expression. To determine whether this effect was in *cis*, we searched for evidence of allelic imbalance (AI) in matched RNA-seq data from the internal validation cohort. Of the cases with sufficient depth and at least one heterozygous SNP in *NFKBIZ*, 24 SNPs in 18 tumours exhibited significant AI favouring the mutant allele. Furthermore, when examining AI of somatic mutations, *NFKBIZ* showed one of the highest frequencies of imbalance (21/33 patients, 64%) compared to other lymphoma-associated genes (Supplementary Figure 7B; Supplementary Table 8).

Mutations affecting predicted structural elements in the 3′ UTR of *NFKBIZ* more commonly exhibited significant AI than those downstream or within the CDS (Fig. 3d). To confirm these observations, we implemented a ddPCR assay that separately quantifies mutant and wild-type *NFKBIZ* alleles and tested mRNA extracted from eight cell lines (Fig. 3e top) and a subset of RNA-seq data from the internal validation cohort. Samples with *NFKBIZ* mutations or amplifications had significantly higher mRNA levels. We confirmed AI favouring the mutant allele in the two cell lines with *NFKBIZ* 3′ UTR deletions (DOHH-2 and SU-DHL-6) and higher I*κ*B-*ζ* protein levels (encoded by the *NFKBIZ* gene) in these *NFKBIZ* mutant lines relative to those lacking such events (Fig. 3e bottom). One cell line (Pfeiffer) which lacked any detectable *NFKBIZ* mutation had elevated *NFKBIZ* mRNA levels relative to un-mutated lines. We suspect this is due to alternative transcriptional regulation, such as *STAT3*, which is mutated in this cell line and suggested to play a role in *NFKBIZ* activation^29,30^.

We then created a series of five UTR constructs, a wild-type fragment representing the well-conserved portion of the 3′ UTR, some of the commonly observed deletions, and two SNVs which affect it. We generated RNA from each of these and, using a combination of methods, found that each mutant altered the RNA structure relative to the wild-type 3′ UTR fragment (Supplementary Figure 8). Further implicating these mutations in modulating the expression of *NFKBIZ*, when placed 3′ to the luciferase CDS, each of the variants caused elevated ectopic expression compared to the wild-type sequence (Fig. 3f).

### Molecular features associated with patient outcome

**Fig. 4:**
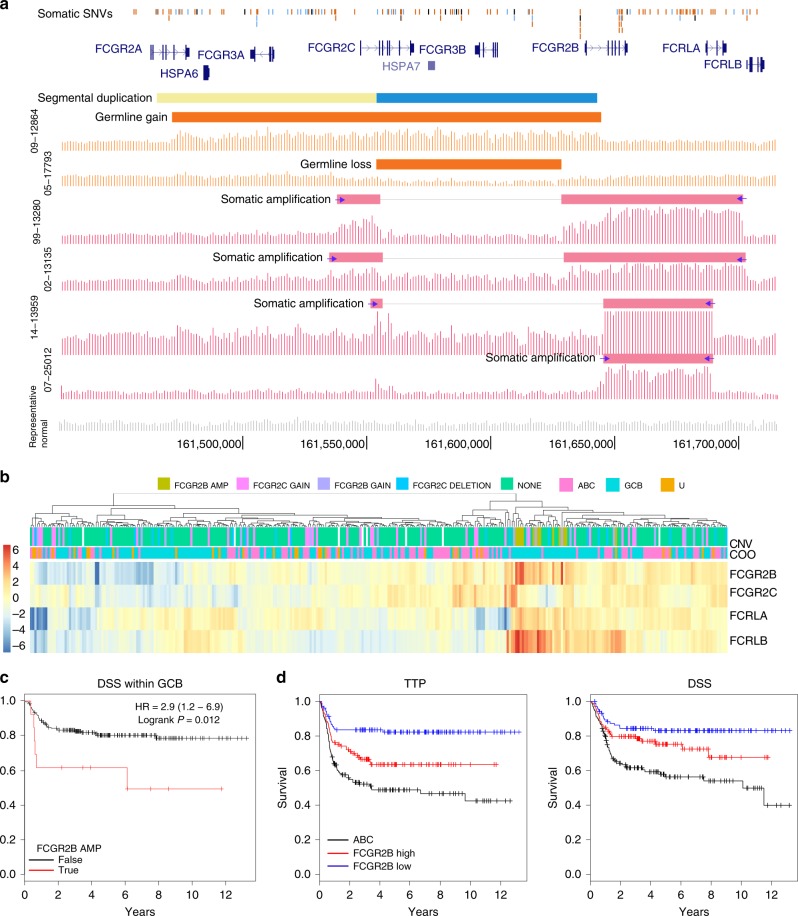
Somatic and germline events affecting the Fc*γ* receptor locus. **a** The genes in the locus are shown with the recent duplication delineated in yellow and blue. Binned read depth from tumours is summarised using vertical bars. Germline CNVs, such as the gain and deletion shown in orange, are common in this region but can be readily distinguished from somatic events in paired analyses. In pink are four examples of somatic *FCGR2B* amplifications. *FCRLA* is completely or partially co-amplified in these. Blue arrows indicate breakpoints identified through visual inspection of data. Horizontal bars delineate the coordinates inferred to be contained within the amplified region. A break in the blue bar corresponding to approximately diploid coverage is indicative of the amplification affecting an allele representing the common deletion CNV. **b** In our validation cohort, we used custom ddPCR and targeted hybridisation capture to infer the presence of gains, deletions, and amplifications. Due to a lack of constitutional DNA for the validation cohort, we are unable to determine the proportion of single-copy gains and losses that can be attributed to common germline CNVs. The expression of each Fc*γ* receptor and *FCRLA* genes in the locus is shown with the cases separated by copy number state. Clustering on the expression of the four genes affected by amplifications groups amplified cases alongside some tumours with gains or no alteration detected, indicating the potential for additional avenues leading to *FCGR2B* over-expression. **c** Although rare overall, cases with the amplification showed a significantly shorter DSS and TTP (*P* = 0.012 and 0.044, respectively; log-rank test). **d** *FCGR2B* expression alone was also significantly associated with DSS and TTP within GCB cases. Specifically, stratifying on median expression or at any cut point above shows that GCB cases with higher *FCGR2B* exhibit significantly shorter TTP (*P* = 4.8 × 10^−3^, log-rank test), although DSS differences require a more stringent cutoff (see also Supplementary Figure 10)

Another striking pattern of mutations identified in this analysis were the focal copy number gains and amplifications affecting the Fc*γ* receptor locus, a complex region of the genome comprising multiple paralogs that have arisen through a series of segmental duplications^31^(Fig. 4a, b). In four genomes, the boundaries of somatic gains could be mapped unambiguously by a combination of read pairing and read depth (Supplementary Figure 9A). The nature of these events and some evidence for fusion transcripts between the co-amplified genes could imply amplification as an extra-chromosomal double minute (Supplementary Figure 9B). It is conceivable, however, that additional structural variants were missed due to a limited ability to uniquely mapping short reads. Establishing the overall incidence of relevant CNVs affecting this locus is also confounded by the presence of common copy number alterations in this region as many of the single copy gains could be explained by germline events in the absence of paired samples. Using a custom multiplex droplet digital PCR (ddPCR) assay, we confirmed the CNVs and identified seven additional examples of amplifications and several additional gains not detected by SNP arrays. Based on these results, the prevalence of amplifications affecting *FCGR2B* was at least 14 out of 451 (3.1%). This is a conservative value including only those events causing changes in copy number beyond those expected from germline CNVs. Further characterisation of these cases with long-read sequencing could allow additional somatic gains detected by this assay to be differentiated from germline CNVs. Among the genes in this region, only the expression of *FCGR2B* (*P* = 0.0357) and *FCRLA* (*P* = 0.0210) were significantly associated with amplification status (generalised linear model, Fig. 4c). Notably, amplifications were mainly found in GCB cases and tumours with an amplification showed strong Fc*γ* receptor IIB protein (CD32B) staining on a tissue microarray, though additional cases with strong staining were also observed (Supplementary Figure 10A).

Several gene-expression, CNV, or mutation-based strategies have been devised to predict outcome in DLBCL^9,10^, with COO and co-occurrence of *MYC* and *BCL2* translocation being the most widely accepted^32^. Translocations involving the Fc*γ* receptor locus and immunoglobulin regions have been described^33,^ but the recurrence of focal amplifications that deregulate *FCGR2B* expression has not been appreciated. Although their prevalence was low, these amplifications were nonetheless significantly associated with inferior outcome in GCB cases. Taking into account the apparent effect of gains on *FCGR2B* over-expression, we hypothesised that elevated *FCGR2B* mRNA and protein was a relevant feature of DLBCL. *FCGR2B* mRNA level was significantly associated with outcome when treated as a continuous variable in a univariate Cox model. We were also able to stratify GCB patients into two groups with significantly different DSS and TTP in univariate Kaplan–Meier analysis spanning a range of thresholds (Fig. 4e, f and Supplementary Figure 10B-C). Using the internal validation cohort, we combined *CIITA* and *IGHG3* mutation status into a multivariate Cox model along with COO and *FCGR2B* expression level and mutation status. Although the trend was preserved for *CIITA*, only *FCGR2B* expression remained a highly significant predictor of outcome in this model (Table 2). This model was highly significant within the external validation cohort and, potentially owing to an enhanced cohort size, was prognostic in the entire cohort including non-GCB cases.

**Table 2:** Multivariate analysis of *FCGR2B* expression on disease-specific survival and time to progression

OS/DSS^a^			TTP		
Cohort, Model	Variable	HR	*p*-value	HR	*p*-value
	*FCGR2B* mRNA > median	2.41	0.156	2.18^*^	5.7 × 10^−3^
BC (GCB only),	*FCGR2B* AMP	2.15	0.140	1.44	0.460
*n* = 210	Any *CIITA* mutation	1.42	0.308	1.68	0.0942
without IPI	Any IGHG1 mutation	1.149	0.747	1.31	0.494
	IPI	3.10^*^	1.03 × 10^−3^	3.07^*^	2.54 × 10^−4^
	*FCGR2B* mRNA > median	1.37	0.387	1.96^*^	0.0397
BC (GCB only)	*FCGR2B* AMP	2.13	0.183	1.48	0.472
*n* = 210	Any *CIITA* mutation	1.29	0.476	1.61	0.143
with IPI	Any IGHG1 mutation	1.23	0.652	1.30	0.535
	*FCGR2B* mRNA > 5	1.52^*^	1.29 × 10^−3^	—	—
Reddy (All)	GCB	0.711	0.0536	—	—
*n* = 530	IPI	2.50^*^	7.55 × 10^−8^	—	—

^a^DSS and TTP was only available only for the BC cohort. Overall survival (OS) was used in place of DSS for analysis of cases from Reddy et al. All *p*-values are from a Cox proportional hazards model

## Discussion

There has been considerable effort placed on developing assays to robustly infer the COO of DLBCL patients, most of which rely on RNA from frozen or formalin-fixed specimens^34^. DNA-based assays may have benefits when RNA is not available and could allow the use of circulating tumour DNA (ctDNA) for this application^35^. Our analysis has revealed numerous non-coding regions with mutations that are associated with COO, and for many, the association is stronger than non-silent COO-associated mutations (Fig. 2). In our validation cohort, we found mutations in *NFKBIZ* in 13.9% of cases, and 18.0% of cases are mutated when CNVs are also considered. These mutations were significantly enriched in ABC DLBCLs (*P* = 4.72 × 10^−10^, Fisher’s exact test), affecting 33.9% of cases in our data. Multiple studies have already attributed a 165-bp region in the UTR that harbours the bulk of the mutations we detected as destabilising elements^27,36^. The observation of AI strongly implicates them in perturbing mRNA turnover, but the functional mechanism is not clear. *NFKBIZ* is one of several genes subject to post-transcriptional regulation by the endoribonucleases Regnase-1 (Reg-1, encoded by *ZC3H12A*) and Roquin^36^. This process involves mRNA turnover and/or sequestration mediated by interactions between these proteins and specific stem-loops in the 3′ UTRs of their targets^37^. Interestingly, *ZC3H12A* was among the novel genes identified herein as recurrently mutated in DLBCL (Supplementary Table 7). *MYD88*, an adaptor protein that is commonly mutated in ABC, is also important for protecting *NFKBIZ* mRNA from this process^38^. Moreover, B-cell receptor signalling, which is active in most ABC DLBCLs, can also promote stabilisation of *NFKBIZ* mRNA via the UTR^27^. Amplifications of *NFKBIZ* in DLBCL cell lines has previously been shown to induce expression of a set of NF-*κ*B target genes in ABC DLBCL^26^. Elucidating the mechanism whereby 3′ UTR mutations impact the NF-*κ*B pathway in DLBCL is highly relevant given the growing list of therapeutic strategies designed to inhibit this pathway directly or by perturbing upstream signalling events. To the best of our knowledge, recurrent 3′ UTR mutations are the first example of a common somatic UTR alteration that can directly increase the expression of an oncogene.

Recent data have implicated common polymorphisms and gene expression differences in tumour tissue in variable response to rituximab, but whether this was due to their effect on *cis* or *trans* interactions remained unclear. In CLL, *cis* interactions of Fc-*γ* receptor on malignant cells is associated with an elevated rate of internalisation of CD32B bound to IgG relative to its other family members^39^. In *trans*, CD32B is directly involved in antibody-dependent cell-mediated cytotoxicity (ADCC), which is triggered by monoclonal antibody-based (mAb) therapies including cetuximab, trastuzumab, and rituximab^40^. We hypothesise that elevated CD32B expression on malignant cells, due in part to the focal amplifications we have identified herein, attenuates the normal immune response to rituximab as seen with alternative isoforms and polymorphic variants of this gene. This was strongly supported by the significantly inferior outcome of *FCGR2B*-high GCB patients treated with R-CHOP (Fig. 4) and is consistent with a smaller study that showed a correlation between CD32B protein staining and outcome in FL^41^. In light of this, alternative immunotherapy approaches may be warranted for this high-risk sub-population. Potential avenues of exploration include Type II monoclonal antibodies directed at CD20 or other proteins, which are not internalised by the same process and thus may be beneficial in these patients, or direct targeting of CD32B alone or in combination with anti-CD20 immunotherapy^42^. Beyond somatic copy number alterations and possibly some influence from germline CNVs, we also identified an elevated level of SSMs in two introns of *FCGR2B* that could promote intron retention and lead to a truncated isoform. As none of the tumours sequenced herein had been exposed to rituximab at the time of biopsy, the effect of these genetic alterations is presumed to also provide a selective advantage in lymphomagenesis, suggesting an oncogenic function for *FCGR2B*. Further exploration of the processes leading to *FCGR2B* over-expression in DLBCL is warranted.

## Methods

### Whole exome sequencing data and analysis

For some of the results, we include included WES data from seven separate published cohorts^10,43–46^. We used the largest cohort, consisting of WES data from over 1000 DLBCL cases^10^ as our external validation cohort. Analysis of the relapsed/treatment refractory DLBCLs and the TCGA cohort was recently described by our group^47^.

### Whole genome sequencing

Patients were diagnosed according to the 2008 WHO classification, as determined by standardised review by expert hematopathologists. Patients were excluded if they had any of the following: primary mediastinal large B-cell lymphoma; primary or secondary central nervous system involvement at diagnosis; a previous diagnosis of an indolent lymphoproliferative disorder; positive HIV serology; a secondary malignancy or major medical co-morbidity that precluded treatment with curative intent. This study was reviewed and approved by the University of British Columbia–BC Cancer Agency Research Ethics Board, in accordance with the Declaration of Helsinki, and all participants were recruited with informed consent.

The genomes included in our analysis represent a compendium of cases from three sources, referred to as the discovery cohort. Namely, we included 39 cases from our previous publication^11^, 41 cases obtained with permission from the ICGC^48^, and another 73 de novo DLBCLs recently sequenced in house. Libraries from the latter were all prepared using PCR-free protocols as previously described^49^. Peripheral blood was used as a source of normal DNA for all cases in the WGS cohort. We performed alignment and detection of SVs, CNVs, and SSMs using matched tumour/normal pairs using standard algorithms and default parameters unless otherwise specified. For SVs, we used Manta and retained variants that pass all default filters^50^. We identified CNVs using Sequenza^51^ and SSMs using Strelka^52^. The ICGC genomes and matched RNA-seq data were downloaded in BAM format and re-analysed using the same methods.

### Targeted sequencing and analysis

We developed a custom gene panel comprising known and candidate DLBCL-related genes and sequenced these regions in tumour DNA from 338 de novo DLBCL patients using a custom hybridisation-capture strategy. This group of samples is described throughout as the internal validation cohort. Of note, a slightly smaller number of cases in this cohort were subjected to RNA-seq (below). First, we sheared genomic DNA to an average of 300 bp using a COVARIS E220 focused ultrasonicator (Covaris) and built libraries from individual DNA samples using the NEBNext UltraII library prep kit (New England BioLabs) according to the protocol provided by the manufacturer. We measured library yields using Qubit (ThermoFisher Scientific) before pooling in batches of 12–16 libraries and mixed with 5 *μ*l of Cot-1 DNA (ThermoFisher Scientific) and 2 *μ*l of xGen Universal Blockers for Illumina platforms (Integrated DNA Technologies). We completely dried each pool in a SpeedVac centrifuge and then resuspended, denatured, and hybridised for at least 4 h with 4 pmol of a panel of xGen Lockdown probes targeting the exons and hotspots of *ADAMTS12*, *ADPRHL1*, *ARID1A*, *ATM*, *B2M*, *BCL10*, *BCL2*, *BIRC6*, *BTG2*, *CARD11*, *CBWD7*, *CCND1*, *CCND3*, *CD58*, *CD79B*, *CREBBP*, *DDX3X*, *DSG4*, *EBF1*, *EP300*, *ETS1*, *EZH2*, *FAS*, *FBXO11*,*FOXO1*, *GABRB3*, *GHDC*, *GNA13*, *GNAI2*, *HIST1H1C*, *HIST1H1E*, *HVCN1*, *ID3*,*IL4R*, *IRF4*, *IRF8*, *KHDRBS2*, *KLHL6*, *KMT2C*, *KMT2D*, *MEF2B*, *MPEG1*, *MS4A1*, *MYC*, *MYD88*, *NFKB1*, *NFKBIA*, *NFKBIE*, *NFKBIZ*, *NOTCH1*, *NR3C1*, *P2RY8*, *PCBP1*, *PDS5B*, *PHF6*, *PIM1*, *POU2F2*, *PTPN1*, *RB1*, *RBM38*, *RFX7*, *RHOA*, *S1PR2*, *SGK1*, *SIN3A*, *SMARCA4*, *SOCS1*, *SPEN*, *ST8SIA1*, *STAT6*, *TBL1XR1*,*TCF3*, *TFAP4*, *TMEM30A*, *TMSB4X*, *TNFAIP3*, *TNFRSF14*, *TP53*, *UBR4*, *USP7*, *ZC3H12A,* and *ZFP36L1*. We supplemented this pool with our own biotinylated baits targeting the *NFKBIZ* 3′ UTR region. We performed targeted enrichment experiments according to the hybridisation capture of DNA libraries using xGen Lockdown probes and reagents protocol (Integrated DNA Technologies)^46,53^. We sequenced enriched libraries on pools on an Illumina MiSeq instrument using PE 150 bp reads and, after alignment with BWA MEM, analysed BAM files for simple somatic mutations (SSMs) using Strelka with an unmatched quasi-normal. Common germline variants with a MAF exceeding 1% in any ExAC population were subtracted, and the remaining variants were annotated the Ensembl Variant Effect Predictor^54^ and converted into the MAF format using vcf2maf (https://github.com/mskcc/vcf2maf). We also inferred SVs with Manta and curated to remove highly recurrent variants likely to represent common variants and recurrent artefacts.

### Gene expression analysis and cell-of-origin determination

All RNA-seq libraries were generated using a strand-specific protocol with poly[A] selection. We used featureCounts (version 1.6.0) to quantify gene-wise expression using all Ensembl gene IDs from the GRCh37.87 release, and set the minimum mapping quality to 10. We normalised gene-wise summary counts for library size using the R package DESeq2, and the resulting normalised expression matrix was used for all subsequent analyses and visualisations. These data were available from 319 cases in the internal validation cohort and from 143 of the genome discovery cohort. We identified 180 cases as GCB using the Bayesian classifier (below) and use this subset for *FCGR2B* differential expression analysis and outcome prediction. Given the requirement of genome-wide information for identifying the effect of genome-wide mutations on expression, we used the data from only the genome discovery cohort for those analyses.

We assigned set of loci previously used to discern ABC and GCB cases to the following 169 distinct Ensembl genes with associated gene names: *A4GALT*, *ADAT3*, *AEN*, *ANKRD13A*, *ANUBL1*, *ARHGAP17*, *ARHGAP24*, *ARID3A*, *ARID3B*, *ASB13*, *AUTS2*, *BATF*, *BAZ2B*, *BCL2*, *BCL2L10*, *BCL6*, *BIC*, *BLNK*, *BMF*, *BPGM*, *BSPRY*, *BTLA*, *C11orf41*, *C13orf18*, *CARD11*, *CCDC50*, *CCDC144B*, *CCND2*, *CCNG2*, *CD47*, *CFLAR*, *CLECL1*, *CLINT1*, *COPB2*, *CREB3L2*, *CSNK1E*, *CYB5R2*, *DCTD*, *DDEFL1*, *DENND3*, *DKFZP434I0714*, *DNAJC10*, *DOCK10*, *EEPD1*, *ENTPD1*, *ERP29*, *ETV6*, *FAM108C1*, *FAM46C*, *FAM53B*, *FLJ32065*, *FLJ42418*, *FOXP1*, *FUT8*, *GNA13*, *GNL3*, *HCK*, *HDAC1*, *HIP1R*, *HOPX*, *HSP90B1*, *ICOSLG*, *IER2*, *IL12A*, *IL16*, *IRF4*, *ITPKB*, *JDP2*, *KCNH8*, *KCNK12*, *KIAA0746*, *KLHL21*, *KLHL5*, *LANCL1*, *LHFPL2*, *LIMD1*, *LMAN1*, *LMO2*, *LOC100129034*, *LOC196415*, *LOC645431*, *LPP*, *LRMP*, *LRRC33*, *MAML3*, *MAPK10*, *MARCKSL1*, *MAST2*, *MME*, *MPEG1*, *MRPL3*, *MYBL1*, *NEIL1*, *NEK6*, *NFKBIZ*, *NIPA2*, *NR3C1*, *OSBPL3*, *P2RX5*, *PAG1*, *PDE9A*, *PDLIM1*, *PFKL*, *PFTK1*, *PHF16*, *PI4K2B*, *PIM1*, *PIM2*, *PLEKHF2*, *PMM2*, *PRKAB1*, *PTK2*, *PTPN1*, *RAB7L1*, *RAP1B*, *RAPGEF5*, *RASGRF1*, *RBM9*, *RECK*, *RILPL2*, *RUNDC2B*, *S1PR2*, *SACS*, *SEPX1*, *SERPINA9*, *SH3BP5*, *SLA*, *SLAMF1*, *SLC1A1*, *SLC33A1*, *SLC38A5*, *SMARCA4*, *SPINK2*, *SSBP2*, *SSR3*, *ST6GALNAC4*, *STAG3*, *STAMBPL1*, *STK17A*, *STS*, *SUB1*, *SULT1A2*, *SYTL4*, *TARS*, *TBC1D27*, *TBL1XR1*, *TCEB3*, *TCF4*, *TCTN3*, *TEX9*, *TGIF1*, *TMEM123*, *TMPRSS6*, *TNFAIP8*, *TNFRSF13B*, *TNKS*, *TOX2*, *TRAM2*, *TTC9*, *USP46*, *VGLL4*, *WNT9A*, *ZBTB32*, *ZFAT*, *ZNF318*, *ZNF385C*, *ZNF511,* and *ZPBP2*. We implemented the Bayesian classifier described previously and calibrated it with all cases having a COO prediction from the Lymph2Cx NanoString nCounter assay^34^. Any case designated as “U” by this assay or with no COO information available were then classified using a cutoff of *P*(*ABC*) <0.9 for ABC and *P*(*GCB*) > 0.9 for GCB. This was only used to assign molecular subgroup to the genome discovery cohort, including the ICGC cases. For the internal validation cohort, all numbers reported were based on Lymph2Cx results rather than this consensus subgroup assignment.

### Allelic imbalance in *NFKBIZ*

Heterozygous SNPs were first identified across all samples in our cohort and annotated using the Ensembl VEP using vcf2maf. For samples with both DNA and RNA sequencing data available, the number of reads supporting the reference and alternate allele of each SNP were quantified using samtools mpileup (version 1.3.1) and a custom script. Intronic SNPs and SNPs with less than 12-fold coverage were excluded from further analysis. Of the remaining positions, any SNP showing evidence of allelic imbalance (AI) was identified by comparing the count of reads supporting each allele between the RNA and DNA BAM files using the Fisher's exact test (Python Fisher package version 0.1.4). Samples with significant AI (*p*-value threshold: 0.05) were further cross-referenced with *NFKBIZ* mutation and SV calls from the targeted sequencing data and copy number information determined using Affymetrix SNP6.0 arrays and OncoSNP. A subset of cases showing AI were selected for validation by ddPCR. AI of somatic mutations was determined through a similar method, using the somatic variant calls obtained from targeted sequencing of the internal validation cohort, as described above. Following false discovery rate correction (Benjamini and Hochberg method), any mutation with *Q* < 0.1 was considered significant. The ratio of patients with AI in each gene was calculated by comparing the total number of patients with at least one mutation in AI to the the total number of patients with at least one mutation overall, for each gene (Supplementary Data 7).

### *NFKBIZ* digital droplet PCR assay

We designed a hydrolysis probe-based assay targeting the *NFKBIZ* 3′ UTR hotspot region. PCR amplicons were chosen to be as small as possible (60–80 bp) and hydrolysis probes with Tm at least 3 °C higher than that of PCR primers. We targeted the 3′UTR with a FAM-conjugated probe and a designed a separate HEX-conjugated probe to target a conserved region of exon 1. This allowed quantification of total transcripts (exon 1 probe) and wild-type transcripts (UTR probe) in cell lines with *NFKBIZ* mutations^55^. We prepared ddPCR reactions in a final volume of 22 *μ*L containing 11 *μ*L of 2X ddPCR Supermix for Probes (no dUTP), a final concentration of 1.0X was used for hydrolysis probes labelled with FAM and HEX fluorophores and a variable amount of input DNA (depending on availability and DNA concentration) and generated droplets using an AutoDGTM System (Bio-Rad). The emulsion of droplets is initially incubated at 95 °C during 10 min in a C1000 TouchTM Thermal Cycler, then followed by 80 cycles of 30 s at 94 °C and 1 min at optimised annealing temperature (58 °C). We determined optimal annealing temperature empirically through a temperature gradient using a DNA sample known to carry an *NFKBIZ* mutation. The emulsion of droplets is incubated at 98 °C during 10 min and kept at 4 °C until analysis in a QX200^*TM*^ Droplet Reader. We analysed the resulting data and assigned clusters using QuantaSoft^*TM*^ software, Regulatory edition (Bio-Rad).

### Cell culture and western blot

Cell lines were cultured in RPMI (Invitrogen) with 10% fetal calf serum (Sigma-Aldrich), except for SU-DHL-4 and SU-DHL-6 which were cultured in RPMI with 20% fetal calf serum, and OCI-Ly10 which was cultured in Iscove‘s modified Dulbecco medium with 10% fetal calf serum. All cell lines were maintained at 37 °C. SU-DHL-4, SU-DHL-6, Karpas422, DOHH-2 and WSU-DLCL2 were purchased from DSZM, Pfeiffer was purchased from ATCC and OCI-Ly10 and HBL-1 were gifts from the Weng lab (BCCRC) to the LCR lab. All cell lines were authenticated by STR profiling. SU-DHL-6. HBL-1 and WSU-DLCL2 were not mycoplasma tested but all others tested negative.

Western Blotting was performed as described^26^ using the Rabbit Polyclonal I*κ*B*ζ* Antibody (TA336346) (Origene) (dilution 1:500) and the Histone H3 Antibody #9715 (Cell Signaling) (dilution 1:1000). Un-cropped western blot is shown in Supplementary Figure 11.

### In vitro *NFKBIZ* 3′ UTR variant effects on protein expression

A fragment of the *NFKBIZ* 3′ UTR was synthesised as a gBlock (Integrated DNA Technologies, Coralville, IA) for the wild-type UTR and four patient-derived mutations: SNV1, Del1, Del2 and Del3 (Supplementary Data 8). The gBlocks were PCR amplified with primers (Supplementary Data 8) to add XbaI sites for cloning. An unrelated region of the 3′UTR was amplified from normal human DNA to act as a control sequence. These PCR amplicons were subcloned into the pGL3-Promoter Vector (Promega) located 3′ to the firefly luciferase translational stop codon. The nucleotide orientation and sequence of constructed plasmids were confirmed by DNA sequencing. For luciferase reporter assays, HEK-293T cells (7 × 10^5^) were seeded in 24-well plates 2 days prior to transfection. Cells were co-transfected with 500 ng of pGL3-pro-NFKBIZ-3UTR (wild-type, mutant or control) firefly luciferase vector and 10 ng of the pRL-TK (Promega) Renilla luciferase vector, using Lipofectamine 2000 (Invitrogen). Assays were performed 24 h after transfection using the Dual-Luciferase Reporter Assay System (Promega). The firefly luciferase signals were normalised to the Renilla luciferase transfection control. Transfections were done in technical and biological triplicates.

### Investigating Fc*γ* receptor copy number alterations

We designed a multiplex ddPCR assay targeting two known genetic polymorphism in *FCGR2A* (rs1801274) and *FCGR2B* (rs1050501) and coding regions of both *BTG2*, also located in chromosome 1, and *ALK* (located in chromosome 2). Probe and primer sequences are shown in supplementary tables. Contrary to standard ddPCR assays, we employed single hydrolysis probes to genotype both SNPs^55^ and leveraged variable final concentrations and two distinct fluorescent dyes for each hydrolysis probe-based assay. ddPCR reactions were carried out in a Bio-Rad QX200 system, using 10–20 ng of tumour-derived DNA and analysed using QuantSoftTM software, Regulatory Edition (Bio-Rad). Copy number gains, losses and amplifications affecting FCGR genes were inferred by calculating and comparing the number of positive droplets for each one of the FCGR genes and those corresponding to each one of the two additional genes used as reference. Our assay targeting rs1050501 co-amplified fragments of both *FCGR2B* and *FCGR2C*. We conducted an independent assay, which replaced the rs1050501 probe with a *FCGR2B*-specific probe that targeted a fixed nucleotide different in exon 3, to differentiate between *FCGR2B* and *FCGR2C*-specific events in a subset of samples. We were then capable to associate common germline deletions and gains with *FCGR2C* and detect focal amplifications of *FCGR2B* with high confidence. Other somatic gains and amplifications in a reduced number of samples involved *FCGR2B* and other Fc*γ* receptor genes.

Further evidence supporting germline and somatic copy number alterations affecting the Fc*γ* region were derived from an independent next generation sequencing experiment relying on targeted hybridisation capture. We built genomic libraries from fresh frozen tumour DNA extracts using the NEBNext UltraII library prep kit (New England Biolabs). These libraries were pooled and enriched using a custom pool of biotinylated xGen lockdown probes (Integrated DNA Technologies) spanning the last two introns of *FCGR2B* and other non-coding regions found to be recurrently mutated in DLBCL. Given the high sequence similarity between paralogs, *FCGR2B*-specific probes also retrieved DNA sequences at equivalent positions for both *FCGR2A* and *FCGR2C*. Enriched libraries were sequenced on a MiSeq instrument (Illumina Inc.) using PE 150 bp reads. Raw FastQ files were imported and analysed using the desktop genomic workbench Geneious (ver. 9.1.5, Biomatters Ltd). Raw reads were aligned using a stringent algorithm that only retained reads displaying high quality mapping scores (â‰¥40) and did not display more than 2% mismatches or indels >3 bp with respect to the reference genome. We then calculated and compared normalised coverage for each gene using for that purpose only annotated regions in the reference genome that enabled an ambiguous assignment of reads. This analysis confirmed elevated *FCGR2B* coverage for those patients suggested to carry focal amplifications by ddPCR and helped corroborate common germline copy number alterations involving a large part or the totality of *FCGR2C*.

### FCGR2B Immunohistochemistry

Tissue microarrays (TMAs) were constructed by using duplicate 0.6-mm cores from diagnostic pre-treatment FFPE tissue^56,57^. Staining was performed on the Ventana platform (Roche, Basel, Switzerland) using routine staining protocols. IHC staining for expression of CD32B (Abcam EP888Y) was independently reviewed by two hematopathologists (G.W.S. and P.F.).

### RNA structural analysis

For SHAPE analysis, WT and other mutant RNA (~1 pmoles) were denatured by boiling them at 95 °C for 3–4 min and then incubated with the folding buffer (Final concentration: 111 mM HEPES, pH 8.0, 6.67 mM MgCl_2_, 111 mM NaCl) for 20 min at room temperature. The folded RNA was then treated with 10 mM NMIA (n-methylisatoic anhydride) for 45 min (5 half-lives) at 37 °C or with clean DMSO for control experiment, followed by ethanol precipitation. The ethanol precipitated RNA was re-dissolved in 10 *μ*l TE (10 mM Tris, pH 7.4 and 0.1 mM EDTA) and mixed with ^32^P-5-labelled primers. Primers were annealed to RNA by incubating the mixture at 65 °C for 5 min and then at 37 °C for 5 min and finally placed on ice for 1 min. SHAPE enzyme mixture (Final Concentration: 75 mM KCl, 50 mM Tris HCl, pH 8.3, 0.5 mM each dNTP, 5.1 mM DTT, 3 mM MgCl_2_) was then added to the RNA-primer annealed mixture. The whole mixture was incubated at 50 °C for 1 min followed by the addition of Superscript III and further incubation at 50 °C for 50 min. After 50 min of incubation at 50 °C, the mixture was treated with 1 *μ*l 4 M NaOH and incubated at 95 °C for 5 min to degrade the RNA. The reaction was stopped by providing equimolar HCl to neutralise the base. Denaturing dye (95% formamide, 1 mM EDTA, and loading dyes) was then added to the mixture and it was heated to 95° for 3 min before loading on 10% denaturing/sequencing gel.

To generate four separate ladders, ~1 pmoles of wild-type RNA was denatured at 95 °C for 3–4 min followed by the addition of radiolabelled primers. Primers were annealed to the RNA by incubating the mixture at 65 °C for 5 min and then at 37 °C for 5 minutes and finally placed on ice for 1 min. SHAPE enzyme mixture (Final Concentration: 75 mM KCl, 50 mM Tris HCl, pH 8.3, 5.1 mM DTT, 3 mM MgCl_2_) was then added to the RNA-primer annealed mixture. 10 *μ*M dNTPs/each were added to the mixture and following amounts of ddNTPs were added to get the separate sequencing ladders for the 4 bases (ddA: 50 ÂµM, ddT: 50 *μ*M, ddC: 100 *μ*M, ddG: 50 *μ*M). The whole mixture was incubated at 50 °C for 1 min followed by the addition of Superscript III and further incubation at 50 °C for 50 min. After 50 min of incubation at 50 °C, the mixture was treated with 1 *μ*l 4 M NaOH and incubated at 95 °C for 5 min to degrade the RNA. The reaction was stopped by providing equimolar HCl to neutralise the base. Denaturing dye (95% formamide, 1 mM EDTA, and loading dyes) was then added to the mixture and it was heated to 95 °C for 3 min before loading on 10% denaturing gel.

For the circular dichroism, each RNA was diluted to a working concentration of 2.5 *μ*M. CD spectra were recorded in a Jasco-810 Spectropolarimeter (Jasco, Easton, MD). The spectra were taken in a quartz cell of 0.5 mm optical path length. The scanning speed was set 500 nm/min with a response time of 1 s. The spectra represent an average of 5 sequential scans over a wavelength range of 200–340 nm, all measured at 22 °C with baseline correction.

### De novo mutation signature discovery

Mutation signatures were discovered using the previously described framework by Alexandrov et al.^58^. We summarised somatic SNVs based on their mutational subtype, 5′ context and 3′ context. This resulted in a mutation catalog matrix of 96 SNV classes for each sample. We performed non-negative matrix factorisation on our mutation catalog to discover mutational signatures within the entire cohort. Signature stability was computed by boostrap resampling over 1000 total iterations (10 iterations in each of 100 cores). The optimal *n*-signature solution, *n*_opt_ which simultaneously maximised signature stability and minimised the Frobenius reconstruction error was automatically selected,

$$n_{\mathrm{opt}} = {\mathrm{argmin}}_n\left( {\frac{{R_n - {\mathrm{min}}(R)}}{{{\mathrm{max}}(R) - {\mathrm{min}}(R)}} - \frac{{S_n - {\mathrm{min}}(S)}}{{{\mathrm{max}}(S) - {\mathrm{min}}(S)}}} \right),$$

where *R* and *S* are the vectors containing reconstruction errors and stability of each *n*-signature solution, and *R*_*n*_ and *S*_*n*_ are the reconstruction error and stability of the *n*-signature solution. To determine matches to known mutation signatures, cosine similarity metrics were computed against the 30 COSMIC mutation signatures. Where more than one signature matched to a single COSMIC signature, the highest similarity match was chosen and the remaining signatures were matched to the next most similar COSMIC signature. Differential exposures of mutation signatures between lymphoma subtypes was performed by the non-parametric Wilcoxon rank-sum test in R programming language and was adjusted for multiple comparisons by controlling false discovery rate.

### Rainstorm analysis

As described in more detail elsewhere^18^, the standard rainfall calculation considers a monotonically increasing set of *N* positions {_*pi*_,…,*p*_*n*_} defining the location of mutations in a single tumour genome (simplified here to a single chromosome). The rainfall plot is a scatterplot of points *S* = (*x*_*i*_, *y*_*i*_) where *y*_*i*_ is given by *y*_*i*_ = log(_*pi*+1_ − _*pi*_) for each *i* ∈{1,2,…,*N* − 1}. The points are often coloured with a scheme that indicates the nature of each mutation such that specific mutation signatures favouring a limited repertoire of substitutions can be visually observed. We note that this plotting method was developed to aid in the study of single cancer genomes^59^. This approach cannot be directly applied to a cohort of patients to highlight areas of the genome that may be affected by mutations more commonly than by chance. Our goal with the rainstorm approach was to overcome this limitation. We developed an extension of the genome wide inter-mutation distance calculation used to highlight local fluctuations in mutation rates within single cancer genomes^59^. Rather than using the distance to the adjacent mutation in the same genome, in our variant, the mean distance to the nearest *n* mutations among unique genomes is used instead. This variation attempts to suppress signal from a limited number of genomes from contributing to the cohort-wide signature.

The Rainstorm algorithm begins with a list of lists, *P* = (*P*_1_, *P*_2_,…,*P*_*g*_) each comprising the monotonically increasing positions from one of the individual somatic mutations in *g* patient genomes. *P*′ is the full (multi-) set of mutation positions *P*_1_∪*P*_2_∪ … ∪*P*_*g*_ for all genomes being considered. We noted a consistent variation in local mutation rate across the genomes included in this analysis. The local trends generally corresponded to the effect of genome replication timing, with regions that consistently replicate late in the cell cycle having a higher mutation rate^60^. We address this by creating a non-overlapping set of bins of equal length *b* (here, *b* = 100 kb) covering the length of the chromosome *l* with the positions contained by the *i*^*th*^ bin represented by *B*_*i*_ and the final bin is constrained to only contain positions ≤*l*.

$$\begin{array}{*{20}{c}} {B_1 = (1,2, \ldots ,b)} \\ {B_2 = (b + 1,b + 2, \ldots ,2b)} \\ \vdots \end{array}$$

The midpoint of each bin is equivalent to the mean of its values, $\bar B_i$. We then determine the mean number of mutations in each of these bins to obtain *μ*, a list representing a course estimate of the cohort-wide local mutation load at the midpoint of each bin.

$$\mu _i = \frac{{\left| {P_i^\prime \cap B_i} \right|}}{b}{\mathrm{for}}\,{\mathrm{each}}\,{\mathrm{bin}}\,B_i$$

We perform local regression on the points $(\bar B,\mu )$ using the loess function in the R statistical computing language. This results in *L*(*P*), a function used here to approximate the mutation rate of each genomic position and adjust for this effect.

For each patient genome we consider a query patient *q*, and we create a |*P*_*q*_| − *by* − *g* matrix *M*^*q*^. Vaguely, we initially populate the entries of *M*_*q*_ column-by-column by listing the differences in nearest pairs of terms in the *P*_*q*_ and the *P*_*j*_ being considered. In particular, for a given patient genome *j* ∈ {1,2,…,*g*} − {*q*}, we pool their set of mutations with those of *P*_*q*_ as a multiset

$$C^j = \{ P_q \cup P_j\}$$

and reorder the terms in increasing order to satisfy $C^j = \{ c_1^j,c_2^j,c_3^j, \ldots ,c_m^j\} ,\mathrm{where}\,\,c_1^j \le c_2^j \le c_3^j \le \ldots \le c_m^j$. Prior to reordering, we store a reference to each index of *C*^*j*^ that derived from elements of *P*_*q*_ and *P*_*j*_. We fill column *j* of *M*^*q*^ by comparing each original position from the query patient with the next highest position in the pooled multiset. Only the comparisons where *c*_*i*_ is originally from *P*_*q*_ are utilised and the *i*,*j*^*th*^ entry in *M*_*q*_ is sequentially populated for each of these values. For example, the *i*,*j*^*th*^ entry of *M*_*q*_ we take the difference between the term at the *i*^*th*^ position originating from *P*_*j*_ in *C*^*j*^ and its adjacent term in *C*^*j*^ etc. This is repeated for all values of *j* with the exception of the case where *j* = *q*, leaving one empty column in *M*_*q*_.

We then apply a numeric sort to every row in *M*^*q*^, which correspond to the original mutation positions in *P*_*q*_. Owing to the convention we use to calculate the pairwise distance *C*^*j*^, this matrix has some useful properties. Mutations that are closer to another mutation in the same genome *P*_*q*_ relative to the comparison genome *P*_*c*_ are completely undefined and thus implicitly suppressed from any further consideration. After the sort, however, the individual rows of *M*^*q*^ no longer relate to the indexes in *P*_*c*_. This new ordering allows us to efficiently find the distance to the nearest mutation in the *k*^*th*^ genome with *k* starting at the genome having the nearest mutation to position *i*, *k* + 1 being the genome with the second-nearest mutation to position *i*, etc. Using this property, we can approximate the density of mutations at every original position in *P*_*q*_ by calculating, for each row *i*, the mean of the values in the first *k* genomes (here, we use *k* = 4). We can increase the specificity of our algorithm to ignore local increases in mutation density in small numbers of patients by increasing *k*. Using the genome-wide mutation rate approximated by *L*(*P*), we then adjust each value for local mutation rate differences after converting to a logarithmic scale. We also correct for the total number of mutations in genome *g*^*q*^, |*P*_*q*_| corrected and use the genome size as a scaling constant.

$$R_i^q = \log \frac{{\left( {\mathop {\sum}\nolimits_{j = 1}^k {M_i^q} } \right)}}{n} + \log \left( {L\left( {P_q} \right)} \right) + \log \left( {\frac{{\left| {P_q} \right|}}{{2.8 \times 10^9}}} \right)$$

This process is repeated for every genome *g*^*q*^ such that we have points (*P*_*q*_, *R*_*q*_) that can be plotted for each patient. The supersets of each, namely *P*′ (defined previously) and *R*′ = *R*_1_∪*R*_2_∪…∪*R*_*g*_, are also used for subsequent analyses. To generate a visualisation that we refer to as a “rainstorm plot” defined by an (x, y) scatterplot (*P*_*q*_, *R*_*q*_) for all *q*∈{1,2,…,*g*} using distinct colour for each *g* with transparency to enhance visibility of overlapping points.

### Doppler Algorithm

The Doppler algorithm delineates mutation peaks using the adjusted cohort-wide inter-mutation distance (as derived above) as input. The values are treated as a frequency variable with index (rather than genomic position) treated as the “time” variable. Wavelet transformations are used in signal processing to decompose a series of spatial or temporal correlated data points. This involves transforming 1-dimensional time series data into 2-dimensional wavelet space along a time scale. Instead of time, we use the index of the ordered set of positions in *P*′, or what we hereafter refer to as “relative position”, or *P*′^*r*^. While the *P*′^*r*^ dimension is the same as in the original time/position series, a new scale is derived from the expanded dimension. When the wavelet transform is applied to time domain data, this scale can be thought as a pseudo frequency, which is highly inversely correlated with frequency but does not have a simple format to transform. If wavelet transform is on frequency domain data, scale can be thought as pseudo time, which is highly inversely correlated to time but do not have a simple format to transform. We treat the relative position on the chromosome as equivalent to time such that the transformation generates a projection of mutation density along the length of the chromosome.

There are two types of wavelet transform: discrete and continuous. For DWT (Discrete Wavelet Transform), the series data are decomposed into an approximation plus multiple levels of details. Approximation and detail decomposition are based on different wavelet base functions. For CWT (Continuous Wavelet Transform), only one basic wavelet function is used, however, the decomposition is based on continuously changing scales and time/locations. We use the CWT implemented in MassSpecWavelet R package^61^ with no prior and using a single variable, *i*.*e*. *R*′ ~ *P*′. Applying the CWT also generates a set of discrete wavelet peaks, each associated with a signal-to-noise ratio (SNR). Manual inspection of the data showed that wavelet peaks are sensitive to small deviations in *R*′ values, leading to overly narrow peaks and fragmentation of some larger peaks. We post-process wavelets individually by chromosome by removing those with a SNR below the 95^*th*^ percentile, based on all wavelets on that chromosome where SNR ≥0. Based on the distribution of *R* values in the chromosome being considered, we define *ϕ* as the 95^*th*^ percentile and *τ* as the 25^*th*^ percentile of *R*. Peaks for which *R* < *ϕ* are removed up-front.

We define the set of positions contained by our *i* individual peaks as *B*, where *B*_*i*_ = (*s*_*i*_, *s*_*i*_ + 1,…,*e*_*i*_). The patient genomes represented within peak *B*_*i*_,

$$g_i^{\mathrm{peak}} = (B_i \cap P_1,B_i \cap P_2, \ldots ,B_i \cap P_g)$$

are a useful metric of the potential biological relevance of mutations in that region to the tumour type represented by the samples. We allow the boundaries of peaks to be refined such that *s*_*i*_ and *e*_*i*_ are adjusted to either shrink or extend the peak size. We allow an extension of the upper and lower boundaries, *s*_*i*_ and *e*_*i*_ outward from the peak of the remaining wavelet positions by considering up to 12 indexes per side. We allow the inclusion of additional mutation positions in this range and stop this process when a mutation is encountered with *R* < *τ*. As well, using the new boundaries, we count the distinct number of patient genomes containing a mutation within the peak boundaries while maximising the outer bounds of *B*_*i*_. If necessary, boundaries are reduced iteratively until the criteria are met or it becomes impossible to meet the criteria for that peak. After this adjustment, we determine the actual mutation rate in each peak in mutations/kb:

$$m_i^{\mathrm{rate}} = 1000 \times \frac{{\left| {P\prime \cap B_i} \right|}}{{\left| {B_i} \right|}}$$

Only the peaks satisfying the two additional criteria $\left| {g_i^{\mathrm{peak}}} \right| \ge 4$ and $m_i^{\mathrm{rate}} \ge 6$ are retained along with the start and end coordinates of the largest |*B*_*i*_| corresponding to the extended or contracted peak meeting this condition.

### Code Availability

The source code for Rainstorm calculation and Doppler peak detection is available on GitHub:  [https://github.com/rdmorin/mutation_rainstorm].

## Electronic supplementary material

Supplementary Information

Description of Additional Supplementary Files

Supplementary Data 1

Supplementary Data 2

Supplementary Data 3

Supplementary Data 4

Supplementary Data 5

Supplementary Data 6

Supplementary Data 7

Supplementary Data 8

## Data Availability

Data available through the European Genome-Phenome archive 146 genome sequence data has been deposited at the European Genome-phenome Archive. Accession number EGAS00001002936. The 1001 genome sequence data^10^ were retrieved from the European Genome-phenome Archive. Accession number EGAS00001002606 [https://www.ebi.ac.uk/ega/studies/EGAS00001002606]
