# Supplementary material for: Genome-wide discovery of somatic regulatory variants in diffuse large B-cell lymphoma
Source: Nat Commun. 2018 Oct 1;9:4001. doi: 10.1038/s41467-018-06354-3 (PMC6167379; doi:10.1038/s41467-018-06354-3)
Supplement: Supplementary file 1 — Supplementary Information [file 41467_2018_6354_MOESM1_ESM.pdf]

Genome-wide discovery of somatic regulatory variants in Diffuse Large B-cell Lymphoma

Arthur *et al*

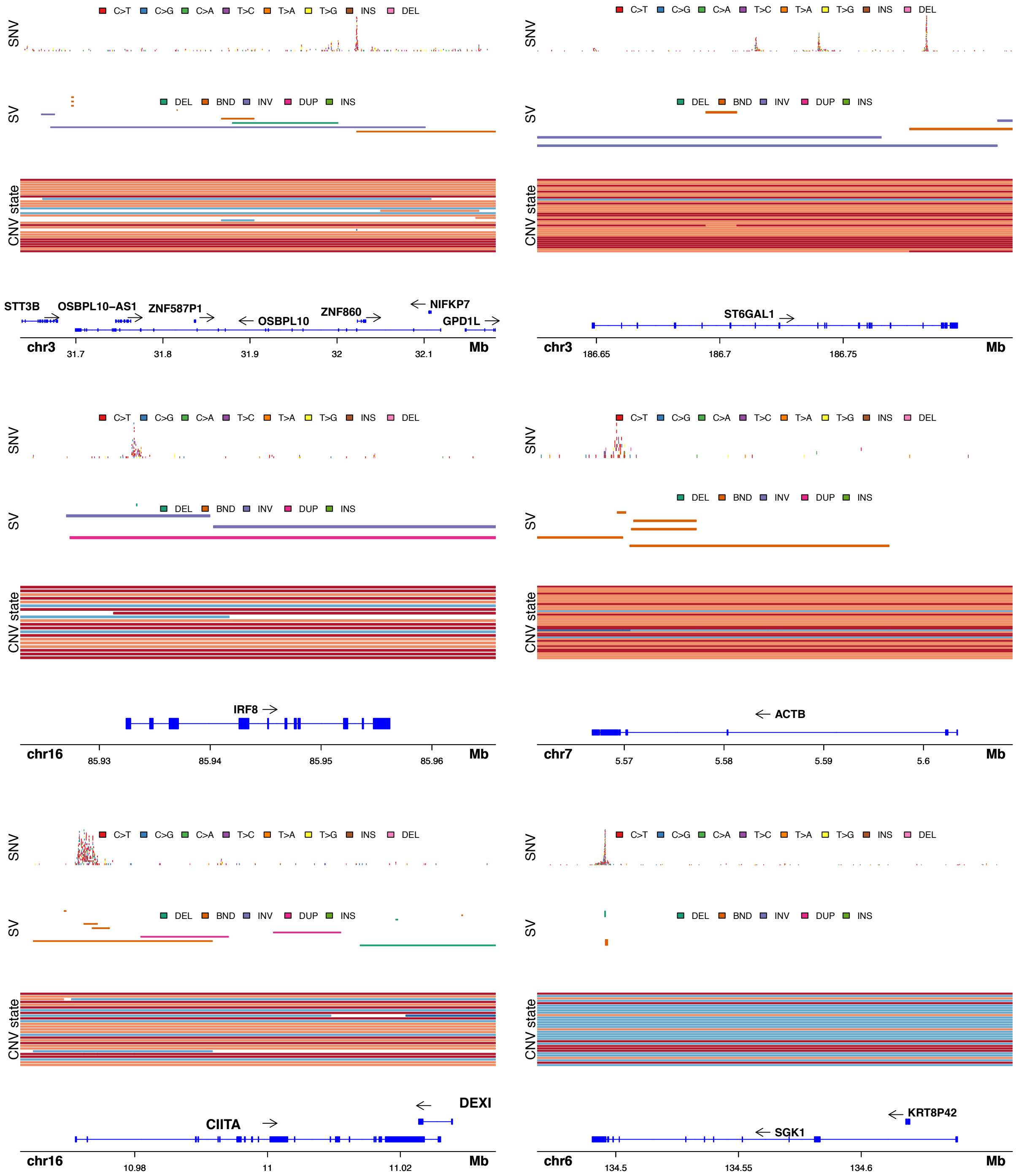

Supplementary Figure 1

Supplementary Figure 1: Examples of genes affected by a variety of mutation types. Six loci with patterns indicative of AID-mediated SHM and structural alterations are shown. Each gene has a peak of mutations downstream of its TSS enriched for C to T mutations. Many such examples were also found to be affected by focal deletions, gains or breakpoints of other SV types within the same region (horizontal coloured bars). In some scenarios, the concentration of mutations could not be readily assigned to a single gene as it affected two genes on opposing strands with a small distance between their TSS (e.g. *OSBPL10* and *ZNF860*). Other affected genes contain peaks at the TSS of only their shorter isoform (e.g. *SGK1*), possibly indicating that is the predominant TSS used in DLBCL, whereas others have multiple peaks including those in intronic regions (e.g. *ST6GAL1*), possibly affecting enhancers or other regulatory elements.

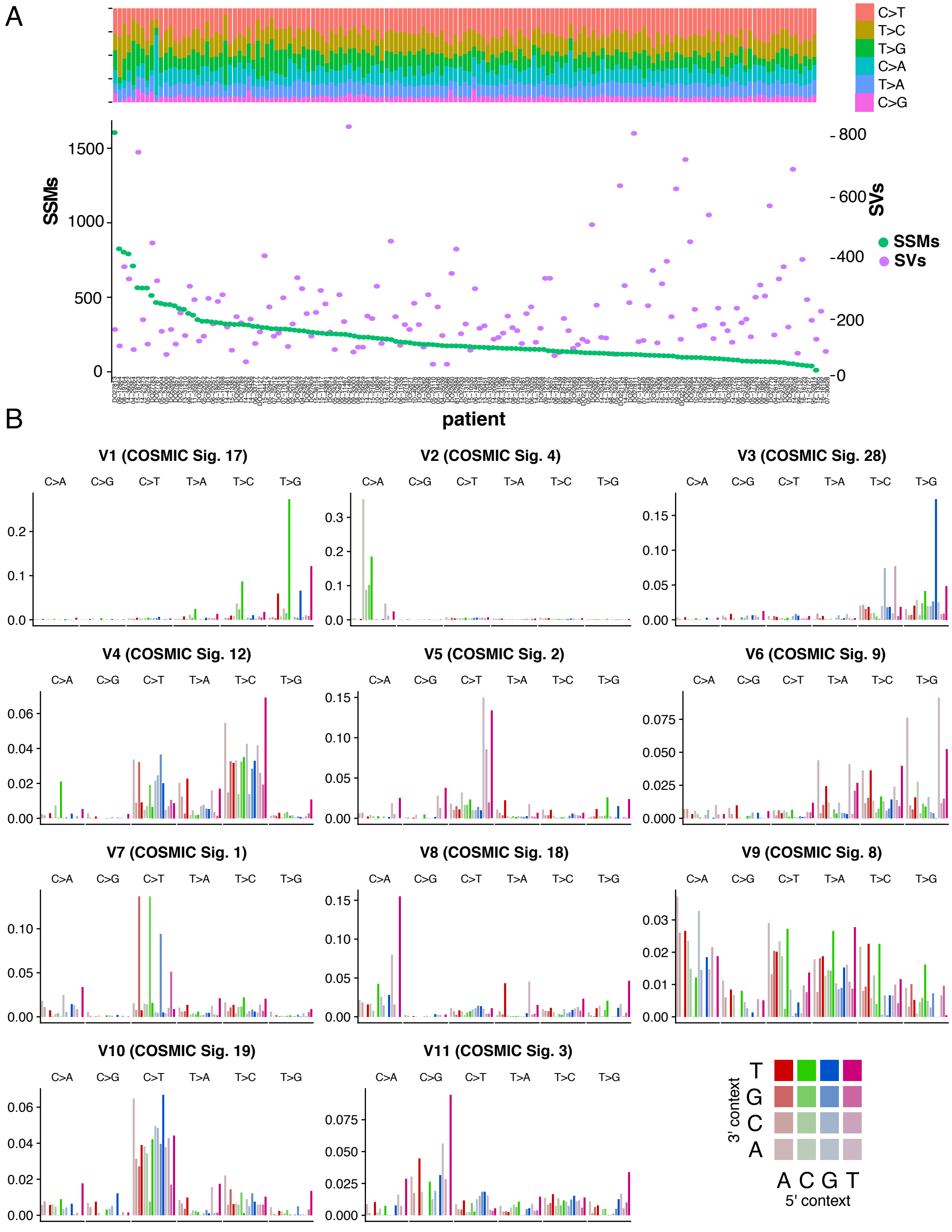

Supplementary Figure 2

Supplementary Figure 2: Mutation signatures inferred from somatic SNVs in 153 DLBCL genomes. (A) Somatic single nucleotide variants (SNVs) were quantified and categorized by base change, collapsing complementary pairs by selecting the pyrimidine reference base. The number of structural variants per sample is also shown. (B) SNVs were further subdivided into 96 classes by incorporating the trinucleotide context of each mutation. *De novo* signature inference yielded an optimal solution of 11 signatures. Each signature has been assigned to the most similar signature in COSMIC, which is indicated in parentheses.

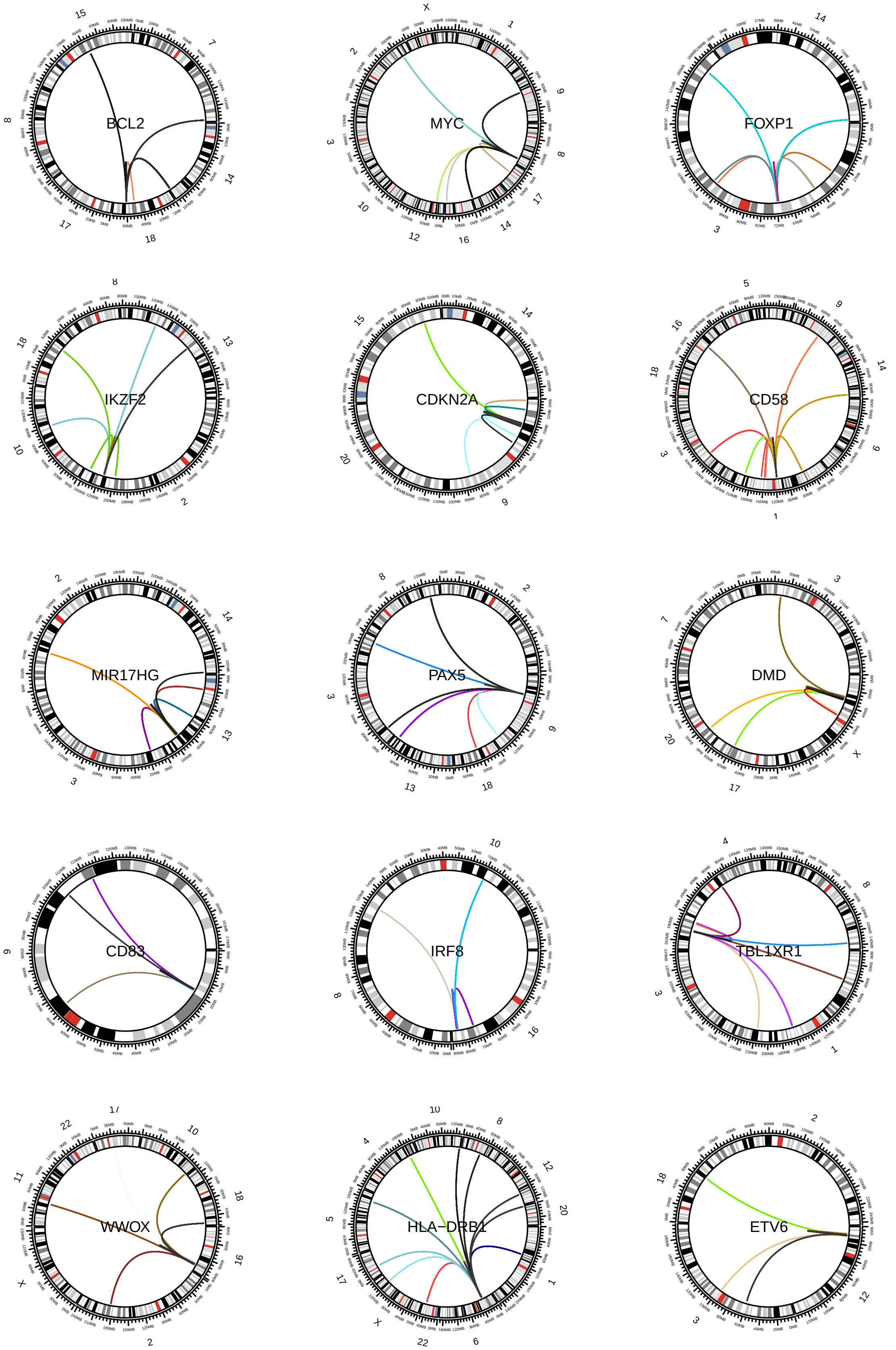

Supplementary Figure 3

Supplementary Figure 3: Recurrence of SVs affecting known or suspected DLBCL-related genes identified by WGS. Based on a targeted sequencing experiment on a subset of these cases, the SVs detected by this approach have a specificity of 95%. SVs that deregulate the expression or impact the function of *BCL2*, *MYC*, *FOXP1*, *CIITA*, *TBL1XR1*, *CKDN2A*, *CD58* and *MIR17HG* have been previously described in DLBCL and other lymphomas. In contrast to *BCL2* and *MYC*, which are commonly rearranged with a limited number of partner loci, the more common trend among the remaining genes was a promiscuous repertoire of rearrangements. The overall diversity and recurrence of SVs affecting *CD58* were notably more common than has been previously reported. Many of the additional examples are known aSHM targets including the *PAX5* locus, *CD83*, and *DMD* which are subject to double-strand DNA breaks due to AID activity.

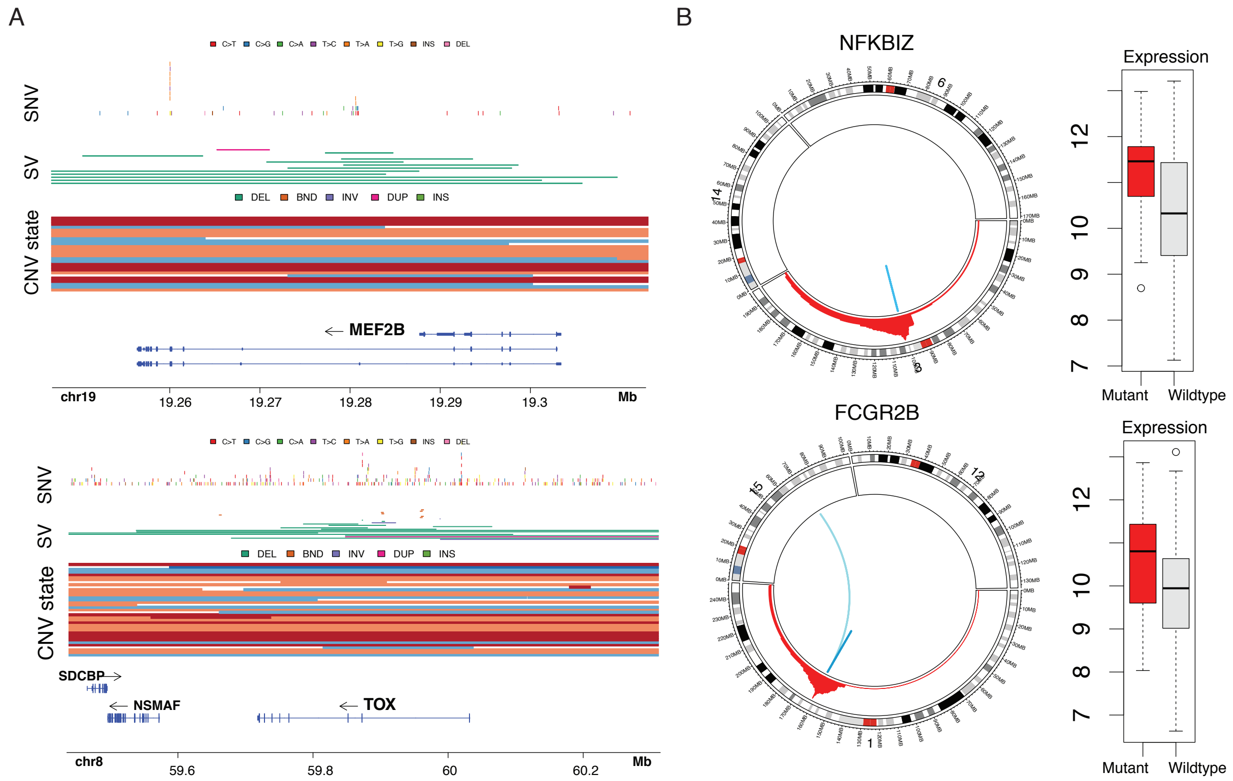

Supplementary Figure 4

Supplementary Figure 4: Structural and copy number alterations indicative of tumour suppressors or oncogenes. (A) Two examples of genes with SV breakpoint and CNV patterns indicating tumour suppressor function are shown. *MEF2B* has two main mutation hotspots. This locus and *TOX* are both affected by multiple focal deletions across the cohort of genomes, whereas amplifications and gains of these loci are rare. (B) Two genes with recurrence of SVs and CNVs showing elevated expression in cases with either mutation type. Only chromosomes involved in at least one SV are displayed for each gene. The red region represents the cumulative number of gains/amplifications encompassing each locus across the cohort of genomes. The expression level of the gene with (red) or without (grey) either a SV or CNV gain affecting the locus is shown (centre). Some of the SVs affecting each of *NFKBIZ* and *FCGR2B* occur in the gene body and may partially disrupt or alter their normal function. SVs involving *NFKBIZ* were all intrachromosomal and included a striking number of small deletions affecting the $3'$ UTR. This region was also enriched for SSMs and was identified by Doppler analysis. Similarly, there were numerous examples of focal CNVs within the Fc-γ receptor locus and a single translocation involving this region.

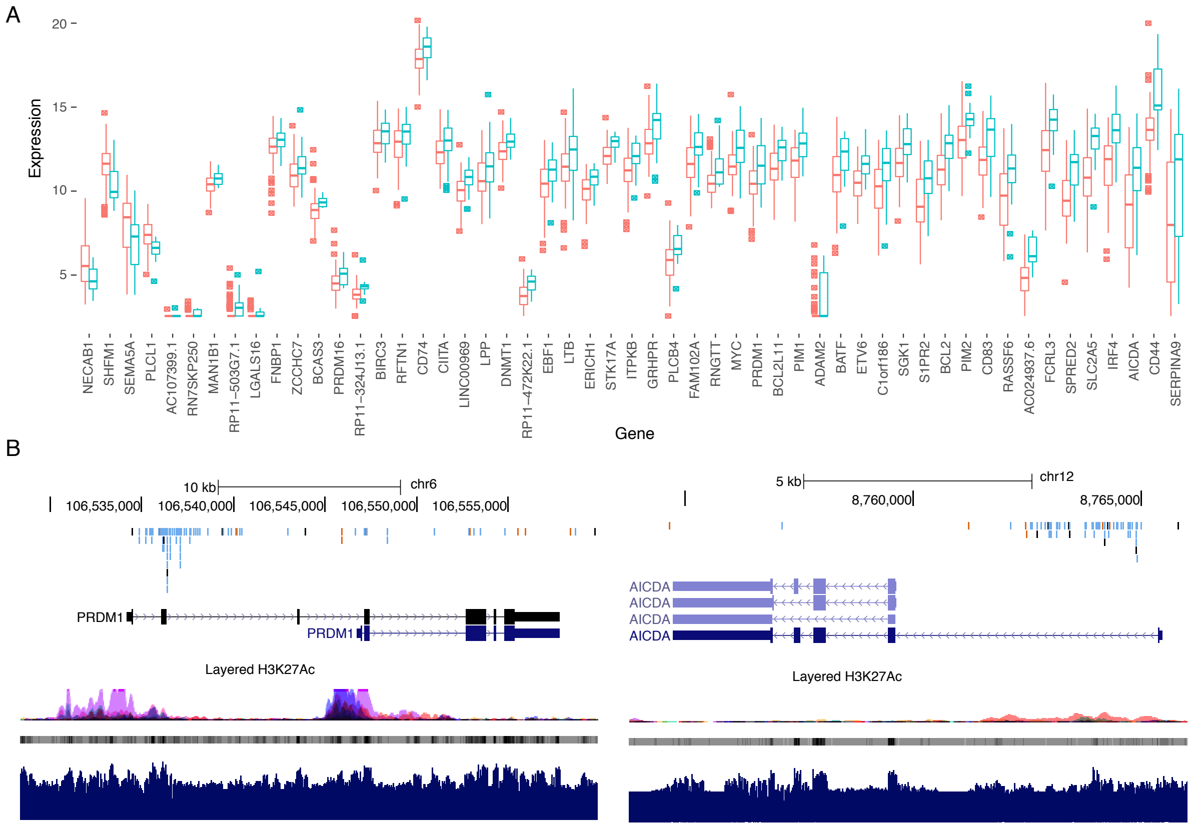

Supplementary Figure 5

Supplementary Figure 5: Genes with differential expression associated with proximal non-coding mutations. (A) This box-whisker plot shows the expression of all genes with significantly different mRNA abundance in cases with and without mutations in a proximal mutation peak identified by wavelet analysis. The bulk of these genes have higher expression in mutated cases (green) compared to cases lacking a mutation in the region (red) and they largely represent known or suspected targets of aSHM. *ZCCHC7* is adjacent to the *PAX5* locus, which contains an enhancer found previously to be recurrently mutated in CLL. These mutations were associated in that study with higher expression of *PAX5*[14]. (B) Two examples of genes affected by aSHM with higher expression in mutated cases are shown. *PRDM1* is a tumour suppressor gene that is commonly mutated and deleted in DLBCL, though here was found to have elevated expression in cases with mutations (mainly intronic). The recurrence of mutations in the first intron identified here, most likely due to aSHM, has not been reported. These mutations were strongly enriched in ABC cases. Given these mutations were associated with higher expression, the bulk of these is unlikely to be functional, though it is conceivable that a subset of aSHM-derived mutations lead to reduced expression. *AICDA*, which encodes the AID enzyme, also had a mutation peak enriched in ABC. The expression of this gene is strongly associated with this molecular subgroup, though these mutations have not been detected by prior studies.

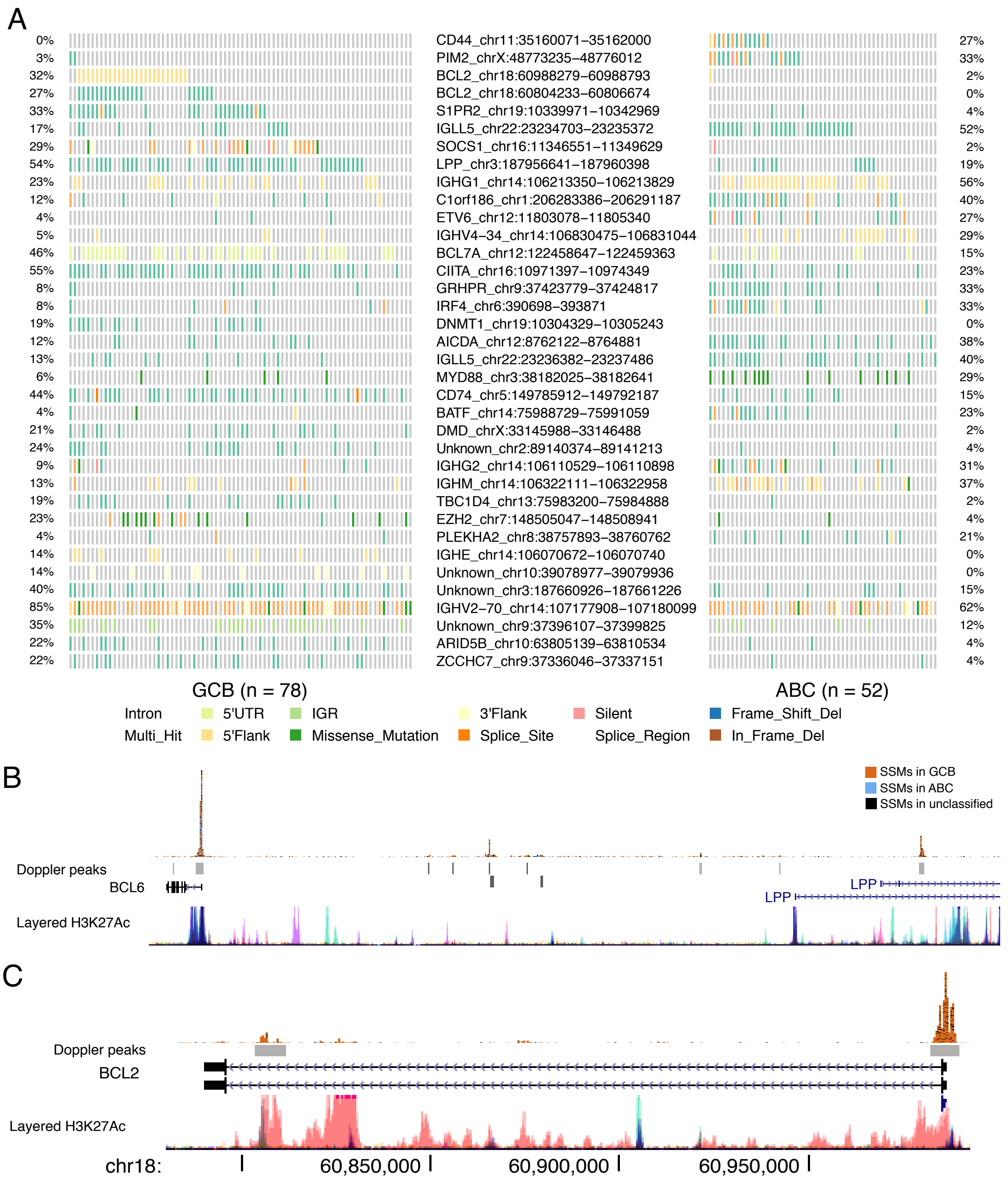

Supplementary Figure 6

Supplementary Figure 6: Coding and non-coding mutations with differential representation between COO subgroups. (A) The top 36 mutation peaks showing significant enrichment for mutations in either ABC or GCB genomes are shown. For each patient (columns), the colour indicates the variant classification for the mutation affecting that locus. The percentage of patients with mutations in each region (based on WGS) is shown on the side for GCB (left) and ABC (right) cases. (B) *BCL2* is a known target of SHM that is typically attributed to translocation to the immunoglobulin heavy chain locus and proximity to the IGH super-enhancer. We detected two GCB-associated mutation peaks in *BCL2* with one spanning the TSS and 5′ UTR and the second residing in the intron. Based on the histone acetylation state (H3K27) determined by ENCODE, both of these are in regions with strong regulatory potential.

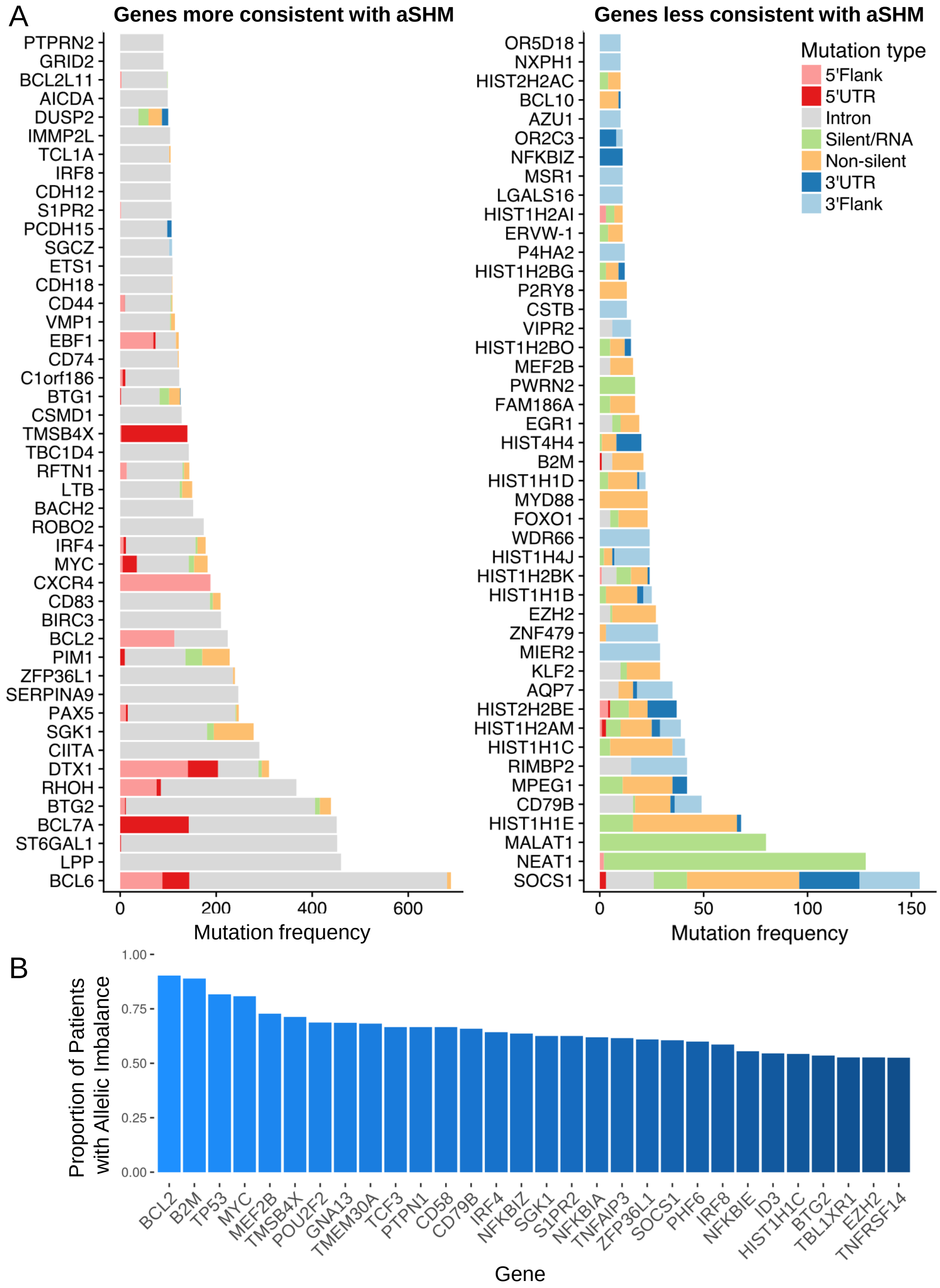

Supplementary Figure 7

Supplementary Figure 7: Mutation peaks and changes in allele expression. (A) Genes with mutation peaks detected separated by pattern. Using the annotation of mutations within the peaks, we determined the most common annotation per patient/peak combination. In most known aSHM targets, this is typically either $5'$ flank, $5'$ UTR or intronic mutations. We identified the genes that had other annotations more common than these as genes less likely to be affected by aSHM. Some of these are RNA genes and thus do not have UTRs (e.g. *MALAT1*, *NEAT1*) and many histone genes, which are small and may have a different pattern due to their length but may nonetheless be affected by aSHM. Others are genes with mutation hot spots such as *MEF2B*, *CD79B* and *EZH2*. Among these genes, *NFKBIZ* appeared distinct $3'$ UTR mutations was the only mutation type within its Doppler peak. (B) We identified somatic mutations which lead to significant changes in RNA abundance by comparing the frequency of somatic mutations identified through DNA sequencing with the corresponding frequency obtained from RNA sequencing. For each gene, the proportion of patients in allelic imbalance (AI) was determined by comparing the number of cases with at least one mutation in AI in that gene, to the total number mutated cases. Genes with less than 50% of cases in AI are not shown.

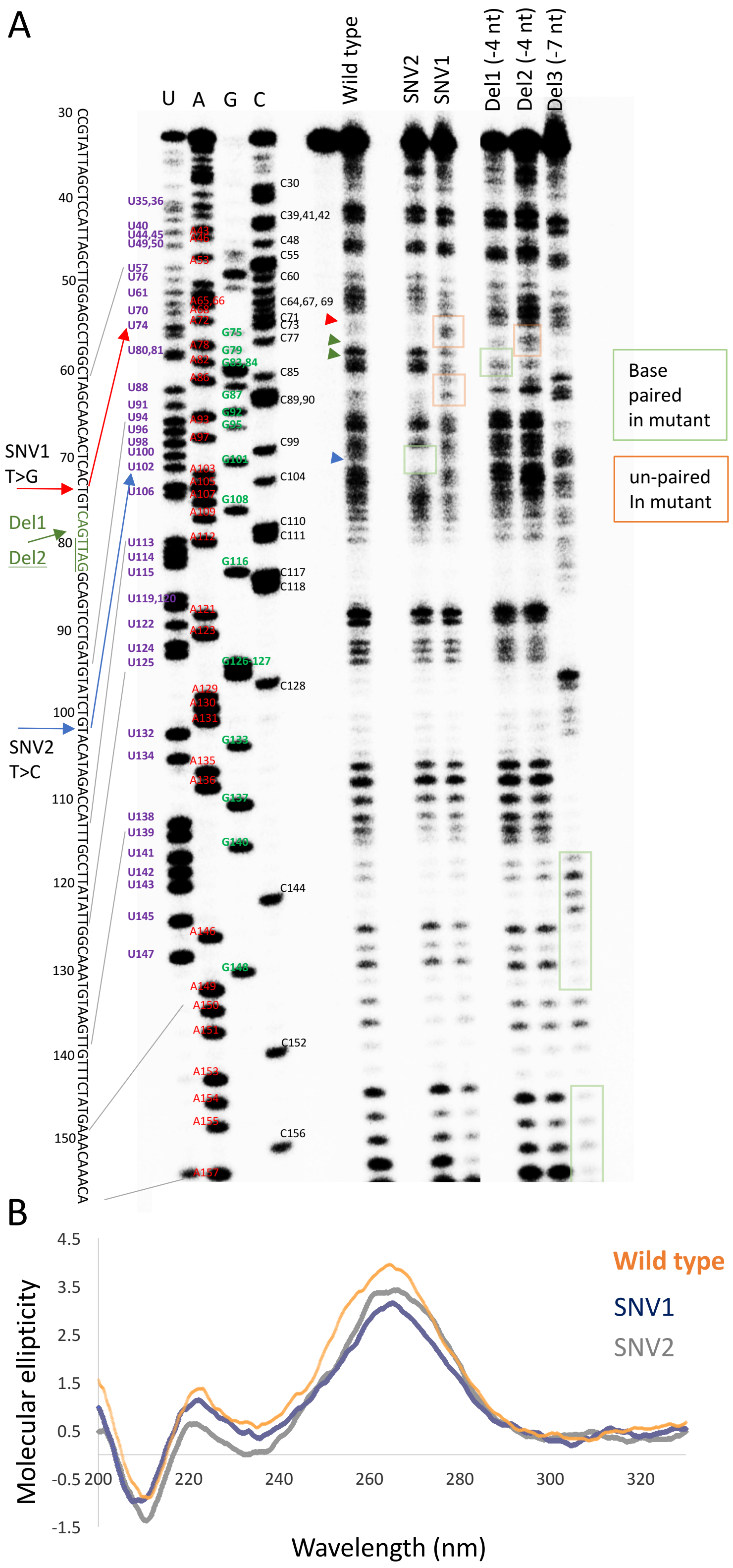

Supplementary Figure 8

Supplementary Figure 8: Structural changes in *NFKBIZ* 3′ UTR induced by common mutations. (A) We performed Selective 2′-hydroxyl acylation analysed by primer extension (SHAPE) on a fragment of the UTR. We generated RNA from synthetic DNA templates representing the wild-type or five mutants including three distinct deletions (Del1-Del3) and two SNVs (SNV1 and SNV2), indicated with coloured arrows on the left of the gel. We prepared a sequencing ladder for each ddNTP using reverse-transcribed RNA from the wild-type template. Each of lanes U, A, G, C represent the complementary ddNTP and are numbered according to the position in the UTR fragment (counting from the 5′). The portion of the sequence resolved on the gel is shown to the left along with numbers corresponding to the position in the RNA fragment. Bands in the remaining lanes indicate the length of truncated RNA caused by modification of exposed nucleotides by NMIA treatment. All RNAs showed reproducible base pairing in some regions. Each of SNV2, Del1 and Del3 exhibited distinct changes in protection, indicating a local change in structure. In SNV1 and Del2, there was de-protection of nucleotides in the same region (orange boxes), which corresponds to the general location of the mutation hot spot (approximately 70-90 in the fragment). (B) We also compared the two SNV mutants to wild-type using circular dichroism (CD). CD spectra for the WT and two mutants resemble the classical ‘A type’ helical duplex, a conformation typical of double-stranded RNA. Given the equal lengths of the three RNAs, CD allows a relative quantitative comparison of Watson-Crick pairs. In agreement with the SHAPE result, each SNV mutation caused a reduction in paired bases relative to wild type with SNV1 having the more extreme effect.

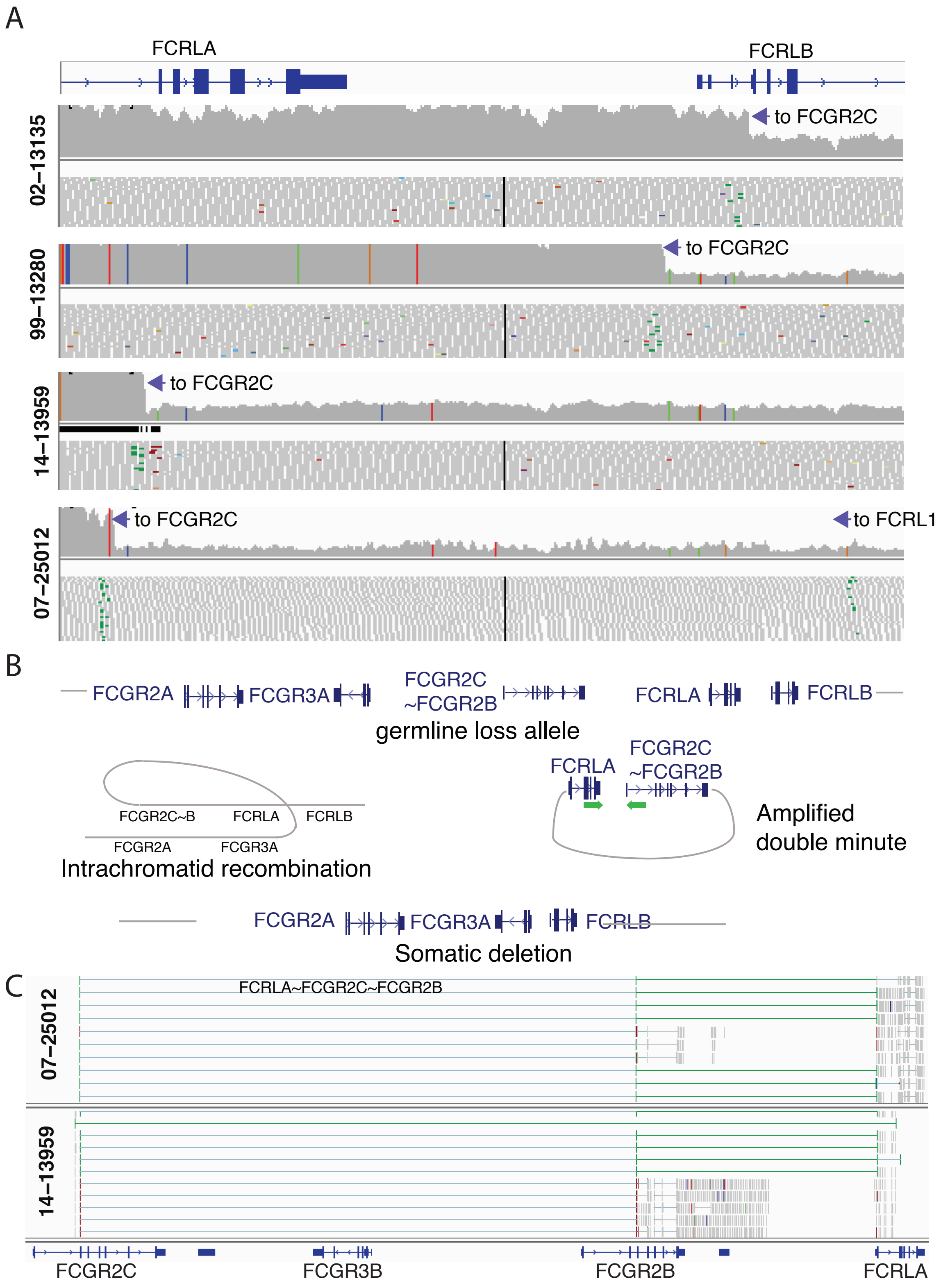

Supplementary Figure 9

Supplementary Figure 9: Details and proposed mechanism of *FCGR2B* co-amplification with *FCRLA*. (A) The distal breakpoints for focal *FCGR2B* amplifications identified in four DLBCL genomes are shown. Each lies within introns of either *FCRLA* or *FCRLB* or the intergenic space between these genes. In patient 07-25012, a second breakpoint representing a copy-neutral inversion was also detected in this region. (B) Because only a single breakpoint pair is detected in most cases, the gain of multiple copies of the locus is consistent with formation of a double minute chromosome containing *FCGR2B* and varying amounts of *FCRLA*. The circular extrachromosomal segment could arise from intrachromatid recombination or aberrant class-switch recombination. Most of the examples of this phenomenon show reduced coverage between *FCGR2C* and *FCGR2B*, which is consistent with this event affecting a germline allele harbouring a deletion and concomitant fusion of *FCGR2C-FCGR2B*. (C) The presence of read pairs in RNA from these cases also supports the presence of a circular double minute. In two patients, reads mapping to *FCGR2C* and *FCGR2B* each have mates that map to *FCRLA*. Green horizontal lines represent reads pairs oriented per the green arrows in (B).

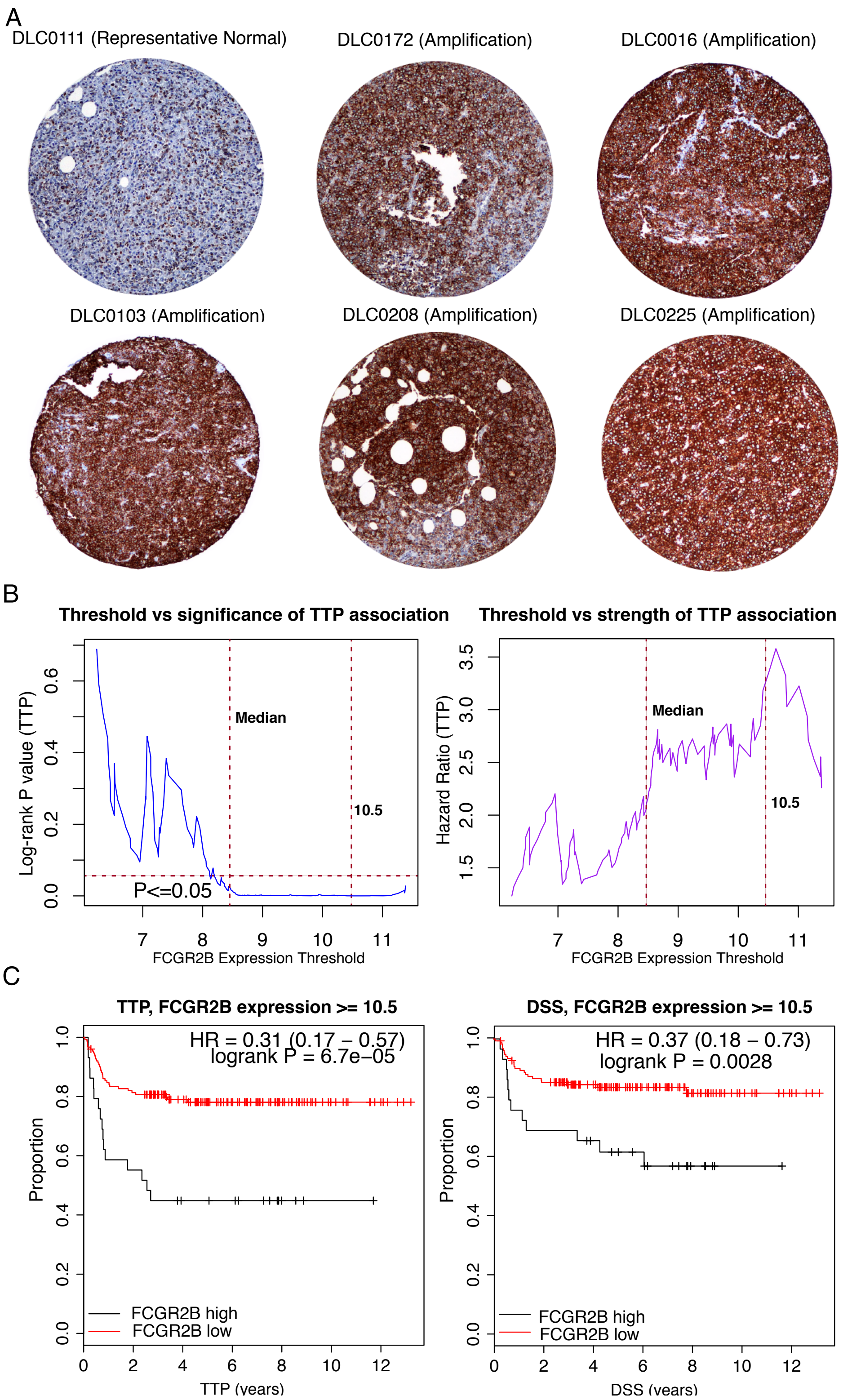

Supplementary Figure 10

Supplementary Figure 10: Determining the relationship between *FCGR2B* mRNA level on patient outcome. (A) Depending on the threshold used to stratify patients, a substantial proportion of *FCGR2B*-high cases have no detectable focal amplification or translocation affecting the locus. We stained a tissue microarray with anti-CD32B to visualize the protein level in patients having and lacking this genetic alteration. A representative example of a *FCGR2B*-normal DLBCL with moderate CD32B staining is shown (DLC0111) alongside five examples that each harbour a somatic amplification. (B) The expression of *FCGR2B* alone was significantly associated with outcome within the GCB cases analysed by RNA-seq. We stratified patients into *FCGR2B*-high and -low strata and tested these two groups for significant differences across a range of thresholds. The P value (left) and hazard ratio (right) showed that any cutoff above the median allowed significant separation of patients on TTP. A similar trend was seen for DSS (not shown). (C) A more stringent threshold of normalized *FCGR2B* expression >10.5 demonstrated a striking separation of cases with very short TTP (left) and DSS (right) reminiscent of ABC DLBCLs.

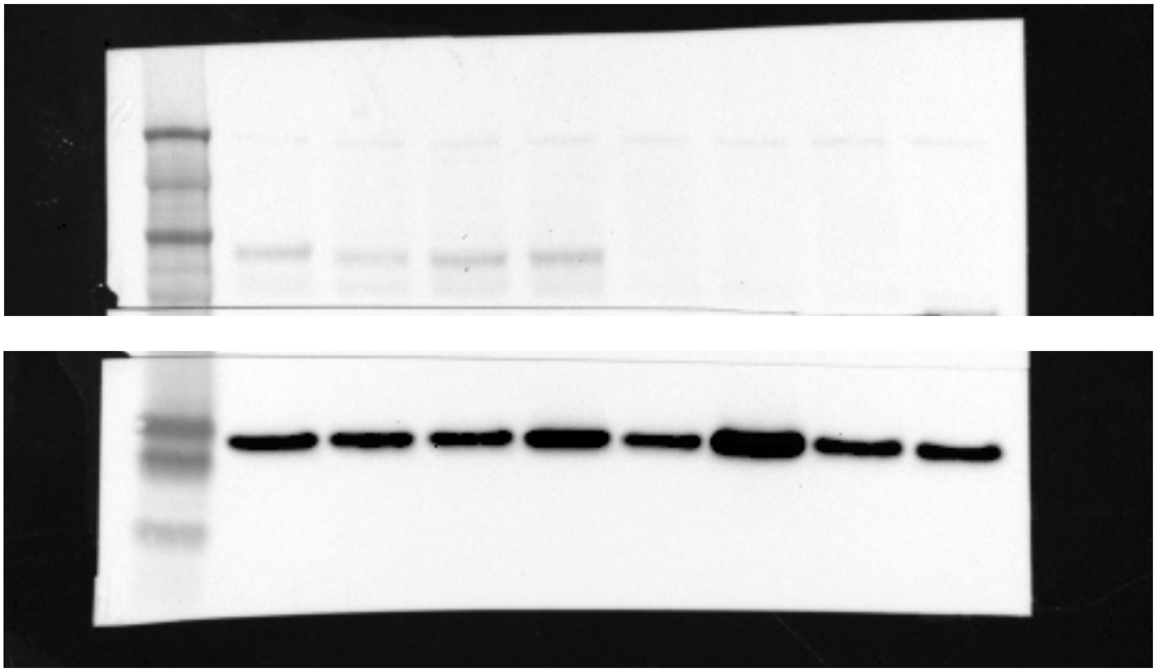

Supplementary Figure 11: Western blot image without cropping. The upper and lower sections are from the same gel but were exposed to different antibodies with the upper showing IκB-ζ and the lower showing Histone protein H3. The middle section of the membrane was not stained with either of these antibodies.
