# Supplementary material for: Genome-wide discovery of somatic regulatory variants in diffuse large B-cell lymphoma
Source: Nat Commun. 2018 Oct 1;9:4001. doi: 10.1038/s41467-018-06354-3 (PMC6167379; doi:10.1038/s41467-018-06354-3)
Supplement: Supplementary file 2 — Description of Additional Supplementary Files [file 41467_2018_6354_MOESM2_ESM.docx]

**Description of Additional Supplementary Files**

File Name: Supplementary Data 1

Description: Sample Overview. Tab 1. Metadata for each patient showing which cohort samples were a part of, what type of sequencing was performed, cell-of-origin and relevant *FCGR2B* and *NFKBIZ* mutation status. Tab 2. Samples with *NFKBIZ* copy number variants (CNVs). Tab 3. Samples with *NFKBIZ* single nucleotide variants (SNVs) and indels. Tab 4. Samples with *NFKBIZ* structural variations (SVs).

File Name: Supplementary Data 2

Description: Single Nucleotide Variants and Indels MAF. Annotated SSMs identified in 153 genomes (in MAF format).

File Name: Supplementary Data 3

Description: Structural Variants MAF. Full set of somatic SVs identified in 153 genomes.

File Name: Supplementary Data 4

Description: Noncoding mutation peaks. Genomic loci identified as enriched for mutations using the Doppler/Rainstorm algorithms after removal of coding SSMs.

File Name: Supplementary Data 5

Description: Full Mutation Peaks. Genomic loci identified as enriched for mutations using the Doppler/Rainstorm algorithms using all somatic SSMs as input.

File Name: Supplementary Data 6

Description: Mutation peaks with differential mutation abundance in ABC and GCB DLBCLs. The result of comparing mutation abundance in these peaks between GCB and ABC genomes is shown. The raw and corrected P values from the Fisher’s exact test and 95% confidence intervals are included.

File Name: Supplementary Data 7

Description: Mutations with allelic imbalance by gene. All mutations identified as having allelic imbalance relative to the variant allele fraction of the tumour were identified. This table lists all genes that were subjected to targeted sequencing and tabulates the number, total and proportion of tumours in which a mutation with AI was identified in that gene.

File Name: Supplementary Data 8

Description: NFKBIZ in vitro gBlock and primer sequences. Tab 1. gBlock sequences of the wild-type and four mutant NFKBIZ 3’ UTR transcripts that were used in *in vitro* experiments. Tab 2. Primers used to amplify gBlocks and the control UTR sequence and add XbaI sites for cloning.
